# Multi-scale entropy analysis of acoustic emission for gearbox fault severity classification

**DOI:** 10.1038/s41598-026-37858-4

**Published:** 2026-02-04

**Authors:** René-Vinicio Sánchez, Yu Liu, Huafeng Qin, Mariela Cerrada, Diego Cabrera, Edwuin Carrasquero, Ruben Medina

**Affiliations:** 1 Universidad Politécnica Salesiana, GIDTEC, Cuenca, 010105 Ecuador; 2 Jiujiang Polytechnic University of Science and Technology, International Shipping Research Institute, Gongqing City, 332020 China; 3 Northeast Electric Power University, Department of Mechanical Engineering, Jilin, 132012 China; 4 Chongqing Technology and Business University, National Research Base of Intelligent Manufacturing Service, Chongqing, China; 5 Chongqing Micro-Vein Intelligent Technology Company, Chongqing, China; 6 Universidad Estatal de Milagro, Research Faculty, Milagro, 091050 Ecuador; 7 South China University of Technology, Mechanical and Automotive Engineering School, Guangzhou, 510641 China; 8 Universidad de los Andes, Engineering, CIBYTEL, Mérida, 5101 Venezuela; 9 Universidad Estatal de Milagro, Sciences and Engineering Faculty, Milagro, 091050, Ecuador

**Keywords:** Acoustic emission, Gearbox fault diagnosis, Multi-scale entropy, Fault severity classification, SHAP interpretability, Condition monitoring, Energy science and technology, Engineering, Mathematics and computing

## Abstract

Acoustic emission (AE) sensors offer significant potential for early fault detection in rotating machinery through the monitoring of high-frequency transients. However, extracting effective features from complex AE signals remains challenging for automated fault severity classification across multiple damage mechanisms. This study investigates multi-scale entropy methods for extracting a computationally efficient set of 16 non-linear information entropy features from AE signals to diagnose gearbox fault severity. Three approaches were systematically compared: Composite Multi-Scale Entropy (CMSE), Hierarchical Multi-Scale Entropy (HMSE), and Composite Hierarchical Multi-Scale Entropy (CHMSE). Experimental data were collected from a spur gearbox test rig operating under controlled conditions, with artificially induced faults representing four damage mechanisms (pitting, broken teeth, root cracks, and scuffing) at nine severity levels each, providing the most granular assessment reported in the entropy-based fault diagnosis literature. Features extracted using each multi-scale method were classified using several classical machine learning models. The CHMSE combined with Random Forests (RF) models achieved the highest classification accuracy (97.37-99.50%), representing a 1-4% improvement over conventional single-scale methods and demonstrating superior performance compared to statistical features and alternative machine learning models. SHAP-based interpretability analysis revealed that generalized entropy measures, specifically Rényi entropy and Tsallis entropy, emerge as primary discriminators across CMSE, HMSE, and CHMSE approaches, with threshold entropy and log energy entropy demonstrating substantial discriminative power when combined with hierarchical decomposition methods (HMSE and CHMSE). Statistical analysis confirmed significant performance improvements (p <0.05) for the hierarchical approaches. These findings demonstrate that CHMSE-based feature extraction enables reliable AE-based condition-monitoring systems for predictive maintenance in industrial gearboxes.

## Introduction

Early detection and accurate classification of fault severity levels are essential in many industries. Rotating machinery, in particular, can be affected by component faults that can lead to catastrophic failure and disrupt the entire industrial process^1^.

Accurate prediction of faults and their severity levels can guide maintenance activities, increasing efficacy and reducing costs. Condition monitoring techniques, which are primarily based on vibration signal measurement and occasionally acoustic emission (AE) signal measurement, are used to perform timely fault detection and classification^2,3^.

Diagnosis of incipient faults in rotating machines is problematic because such faults tend to be buried by time variations of the process related to machinery equipment^4,5^. Attaining this goal is possible by using efficient monitoring systems. Condition monitoring is most commonly performed based on vibration signal measurement due to the availability of tools for signal acquisition and analysis. However, the use of AE signals has also been investigated, and several advantages over vibration signals have been reported^6–8^.

Rotating machinery is used across different industries and includes motors and other components, such as roller bearings and gearboxes. Gearboxes are particularly useful for transmitting high torques in small spaces. A gearbox typically consists of several devices, such as gears and roller bearings^9,10^. The common types of faults in gearboxes are cracks, broken teeth, wear, pitting, and scuffing^11–14^. Contact stress is the leading cause of surface pitting and wear. This type of defect causes transient events when the faulty tooth is in mesh, which can lead to the development of cracks and tooth breakage^11,15–17^.

Early detection of pitting faults is typically performed using condition monitoring techniques based on vibration signals^18,19^. However, acoustic emission (AE) signals are also feasible for condition monitoring. AE signals consist of acoustic waves radiated by solids when their internal structure changes, such as through crack formation or plastic deformation due to aging, temperature gradients, or external mechanical forces.

In rotating machinery, AEs consist of transient elastic waves generated by the interaction of two media in relative motion. For example, AE is generated in rotating machinery by impact, friction, cyclic fatigue, or material loss^20–22^. AE signals can characterize defects earlier than vibration signal analysis. However, their main limitation is attenuation when the sensor is located in the case of the faulty component^10,23,24^. Additionally, AE signals are highly non-stationary and non-linear, making their analysis difficult. Such signals represent a non-linear system^25,26^, therefore, non-linear dynamics techniques and chaos theory can help investigate them.

Deep learning remains underutilized for fault-severity classification, particularly for acoustic emission (AE) signals. Recent studies, such as the ones in^27^ and^28^, classify milling and gear faults by fusing vibration and AE data or by using MEMS AE sensors. Both approaches handle high-frequency AE data by converting signals into time-frequency representations before feeding them into ResNets or multi-input CNNs. Other architectures, such as 1D-CNN and Bi-LSTM, have been combined for mineral analysis^29^, but this method has not yet been applied to rotating machinery. The research reported in^30^ demonstrates success with 1D-CNNs at sub-standard vibration sampling rates using 120-sample windows, but this methodology is not directly transferable to AE. Architectures like SART-1DCNN^31^ have been proposed for pipeline leak detection; they often rely on relatively short windows (e.g., 1024 samples) without specifying the sampling frequency, leaving open the question of how they would scale to true 1 MHz AE monitoring. Similarly, works using 1D-CNN with channel attention for tool wear^32^ often operate at lower sampling rates, where 1D-CNNs remain computationally manageable. Zhang et al.^33^ proposed a variable-pooling multi-scale CNN (VPMCNN) that addresses feature weighting limitations in traditional pooling layers and achieves 98.02% accuracy for bearing fault classification. By introducing weighted pooling channels and multi-scale fusion modules, their approach demonstrates the potential of automated feature learning from high-frequency AE signals. However, deep learning approaches like VPMCNN face several critical limitations for industrial deployment. First, CNN-based methods operate as black-box models, providing limited interpretability regarding which signal characteristics drive diagnostic decisions^30–32^. Second, these approaches typically require extensive labeled training data^33^ and substantial computational resources for training and inference, limiting their applicability when fault data is scarce or edge computing deployment is needed. Third, while the VPMCNN addresses fault type classification, it does not tackle the more challenging problem of multi-level fault severity assessment, which is essential for prognostic maintenance decision-making, and remaining useful life (RUL) prediction.

Graph-based fault diagnosis methods have also emerged as powerful alternatives for multi-sensor rotating machinery monitoring^34–36^. In particular, Li et al.^37^ proposed the Dynamic Causal Mechanism Learning Diagnostics Framework (DCMLDF), which integrates temporal encoding, adaptive causal graph inference, and structure-aware prediction to model fault propagation pathways in complex rotating machinery. By learning directed causal dependencies between sensor channels using graph neural networks, their framework achieved 97.95% accuracy in bearing fault classification and demonstrated improved robustness across varying load conditions. However, critical limitations prevent direct application to AE-based gearbox severity classification. The DCMLDF was designed for low-frequency vibration signals sampled at conventional rates, whereas AE monitoring requires 1 MHz sampling to capture high-frequency transient bursts. Furthermore, the multi-layer graph convolutional operations scale quadratically with the feature dimension, making their application to extended AE windows computationally prohibitive. Additionally, the framework addresses fault type classification rather than multi-level fault severity assessment, and its multi-channel interpretability advantage is lost in single-sensor AE monitoring configurations.

Advances in few-shot bearing fault diagnosis have introduced metric-encapsulation (ME) frameworks combining Transformer architectures with distance metric learning^38,39^. These approaches employ multi-scale feature extraction with global Transformer branches for capturing context, and local Mahalanobis distance-based metric branches for class-aware separation. By learning class-specific covariance structures rather than using simple Euclidean distances, metric-learning approaches can distinguish between fault classes with limited training data.

While non-linear dynamic features based on phase space reconstruction (approximate entropy, sample entropy, fuzzy entropy) have proven effective for vibration analysis^25,40,41^, their application to high-frequency AE signals is limited by computational complexity. These methods require distance calculations between high-dimensional phase-space vectors, as in Eq. 1. This computationally intensive operation scales poorly with the large time-series windows typical of 1 MHz AE sampling. Consequently, this study employs information-theoretic entropy measures–including wavelet packet entropy, spectral entropy, and generalized entropy measures (Rényi and Tsallis)–which avoid explicit phase-space distance calculations while maintaining sensitivity to signal complexity and non-stationarity. These computationally efficient features enable analysis of extended AE signal windows without prohibitive processing overhead, making them suitable for practical industrial implementation.

Non-linear vibration signal analysis in rotary machines has been performed using fractal and chaos techniques^25,40–45^. Non-linear features have also been used to analyze acoustic emission signals^46,47^. In particular, information-based entropy features have been used to detect cracks in materials using AE signals^46,48^. This feature type has also been used to diagnose inter-shaft bearing faults^49^. Similarly, approximate entropy analysis has been applied to AE signals to detect defects in roller element bearings^50,51^. In^52^, they have combined the variational mode decomposition of AE signals with energy entropy for early fault detection in a gearbox. A review of the use of machine learning algorithms in acoustic emission applications, examining their advantages and limitations, is reported in^53^.

Multi-scale approaches for fault detection in rotating machinery have been widely reported in the literature^54,55^. Chen et al.^56^ proposed a multi-scale approach for classifying faults in roller bearings and gearboxes using vibration signals. The method involves preprocessing the vibration signals using Intrinsic Time Decomposition (ITD) and selecting the optimal component with the highest kurtosis. Subsequently, the improved multi-scale amplitude-aware permutation entropy (IMAAPE) is employed to extract features, which are then used by a multi-class relevance vector machine (mRVM) for fault classification. The method achieved 100% accuracy in classifying five conditions of a gearbox test rig.

Zhang et al.^57^ presented a method for fault diagnosis in a wind turbine gearbox using vibration signals. The method begins by decomposing the vibration signal using the improved variational mode decomposition (IVMD) algorithm, which estimates the optimal VMD decomposition parameters. After decomposition, the Intrinsic Mode Function (IMF) with the minimum energy loss coefficient (ELC) is selected for feature calculation. The features are calculated using time-shift multi-scale sample entropy (TSMSE), and classification is performed using a variant of the SVM algorithm, the sparrow search algorithm-based support vector machine (SSA-SVM). The algorithm was validated on a wind turbine test rig with four fault conditions, achieving good classification results.

Wu et al.^58^ introduced the concept of Composite Multi-scale Entropy, equivalent to TSMSE, and applied it to classify roller bearing faults. Their method achieved higher accuracy than the Multi-Scale Entropy (MSE) approach.

Wei et al.^59^ proposed a refined composite hierarchical fuzzy entropy method for early fault detection in planetary gearboxes. Their method demonstrated effectiveness in identifying early-stage gearbox faults.

Such Multi-scale approaches have proven effective for fault detection and diagnosis in rotating machinery since they offer the advantage of capturing fault-related information across multiple scales, thereby improving accuracy and robustness in fault identification. However, most reported research has focused on vibration signals, with limited studies employing acoustic emission signals for multi-scale analysis.

A review of published literature reveals a critical research gap: while hierarchical entropy methods have demonstrated effectiveness in vibration-based fault diagnosis^59–61^, their application to acoustic emission signals remains unexplored. Specifically, all existing implementations of hierarchical entropy decomposition methods, including Hierarchical Fuzzy Entropy (HFE), Refined Composite Hierarchical Fuzzy Entropy (RCHFE), Hierarchical Dispersion Entropy (HDE), and Composite Hierarchical Multi-scale Sample Entropy (CHMSE), have been exclusively demonstrated on low-frequency vibration data. The absence of hierarchical entropy applications to high-frequency AE signals represents a significant opportunity, particularly given AE’s documented advantages in early fault detection^6–8^. Furthermore, existing severity classification studies typically address 3–4 severity levels^62^, which limits their utility for early-stage fault detection and prognostic decision-making.

These research gaps motivated this study. We hypothesized that composite hierarchical multi-scale entropy combined with information-theoretic features could overcome these limitations and achieve reliable AE-based fault severity classification across multiple damage mechanisms and severity levels.

The main contributions of this work are:

- First application of Composite Hierarchical Multi-scale Sample Entropy (CHMSE) to fault diagnosis in rotating machinery using acoustic emission signals, extending the applicability of hierarchical entropy methods to a new signal modality with distinct physical characteristics.
- Classification of nine severity levels per fault type, representing the most granular severity assessment reported in the entropy-based fault diagnosis literature.
- Systematic comparison of CMSE, HMSE, and CHMSE under identical experimental conditions, providing evidence-based guidance for method selection.
- Identification of a computationally efficient feature set of 16 non-linear entropy measures that balances classification accuracy with computational feasibility for industrial implementation.
- Integration of (SHapley Additive exPlanations) SHAP-based interpretability analysis that identifies a consistent set of generalized entropy measures, Rényi entropy, and Tsallis entropy, as primary discriminators for fault severity classification across all multi-scale approaches. The analysis further reveals that threshold entropy and log energy entropy are particularly important when combined with hierarchical decomposition methods (HMSE and CHMSE). By revealing which physics-informed entropy features most strongly influence predictions, this analysis enhances transparency and addresses the black-box limitation of deep learning approaches, facilitating trust in safety-critical industrial applications.

## Related work

### Reconstruction of the phase space

Analysis of non-linear dynamical systems is a complex task. However, it is possible to recover an equivalent dynamics of such systems from recorded time domain signals using the phase space reconstruction techniques. Such reconstruction is based on Taken’s embedding theorem reported in^63^ for univariate time series and in^64^ for a multivariate version.

Concerning the univariate phase space reconstruction, given a signal denoted $\textbf{x}$ including *N* samples denoted $\textbf{x}=[x_1, x_2, x_3,..., x_N]$ that represents the projection of a segment of the system trajectory. According to Taken’s embedding theorem, the phase space reconstruction generated by time-delayed ($\tau$) components of $\textbf{x}$ is expressed as:

1 $$\begin{aligned} \textbf{X}_n=[\textbf{x}_n,\textbf{x}_{n+\tau }, \textbf{x}_{n+2\tau },...,\textbf{x}_{n+(m-1)\tau }], \end{aligned}$$

where *n* varies from 1 to $M=N-(n-1)\tau$ with *M* representing the number of points or states of the phase space and *m* is the dimension of the reconstructed space.

### Non-linear entropy

Several measures of regularity for time series are recorded from non-linear dynamical systems. Examples of such measures are the approximate entropy (ApEn)^65,66^, the sample entropy (SampEn), which is obtained with modification of the ApEn Algorithm^67^, and the fuzzy entropy (FuzzyEn)^68^. These entropy measures are based on calculating distances between vectors spanning the phase space reconstruction in Eq. 1. In addition, other non-linear features also rely on the calculation of distances between vectors of the phase space reconstruction, such as the correlation dimension (CD)^69^ and the most prominent Lyapunov exponent (LLE)^70^. All these types of features are computationally intensive. Consequently, their application to large time series vectors could take too long to analyze.

While some non-linear entropy measures (ApEn, SampEn, FuzzyEn) rely on phase space reconstruction (Eq. 1) and distance calculations, the information-based entropy features selected in this study (Table 1) avoid these computationally expensive operations. We present the phase space framework for completeness and to contextualize our feature selection rationale.

#### Permutation entropy

Although the permutation entropy implicitly relies on the phase space reconstruction, its computational cost is lower than that of the aforementioned measures because it does not require calculating vector distances. The permutation entropy requires partitioning the phase space into non-overlapping cells^71^. A more detailed description of the calculation methodology for the permutation entropy is presented in^72^. An efficient method for permutation entropy calculation is reported in^73^.

### Information based entropy measures

The information entropy was proposed by Claude Shannon^74^ in the context of theoretical communication modeling. Entropy measures can be calculated based on time or on the time-frequency representation of the time series, such as wavelet packet entropy and power spectral entropy. However, other measures, such as the Tsallis and Rényi entropy, are also considered in this research and represent two generalizations of the Shannon entropy^75^.

#### Wavelet packets entropy

When dealing with discrete-time signals represented as vectors, the entropy concept has also been applied to select the best basis for orthogonal wavelet packets^76^. The authors show that several entropy measures can be estimated from the wavelet packet decomposition of the signal. Such entropy measures are the Shannon Information Entropy, the Normal Entropy, the Log energy Entropy, the Threshold Entropy, and the Sure Entropy.

#### Power spectral entropy

The power spectral entropy (PSE) quantifies spectral complexity in the frequency domain from a power spectral density perspective^77^. This type of feature has been used for fault detection in gearboxes and roller bearings^78,79^. The power spectral density is calculated from the time series, normalized, and converted into a power spectral density distribution. It is computed as a time-frequency representation; the power spectral entropy is computed for each time frame and forms a vector denoted $\textbf{S}$.

### Multi-scale entropy

The multi-scale entropy was proposed in^80^. The method enables the estimation of non-linear entropy (and its variants) at multiple temporal resolutions of the signal. The approach was first applied to biological signals, and later, it was applied to the problem of roller bearing faults detection in^81,82^, and bearing faults classification using permutation entropy in^83^. The calculation of multi-scale entropy can be performed for approximate entropy, fuzzy entropy, sample entropy, corrected conditional entropy, permutation entropy, or any other entropy-based or statistical feature. For the univariate time series of length *N*, denoted $\textbf{x}=[x_1, x_2, x_3,..., x_N]$, it is possible to construct a set of coarse-grained time series denoted $\{y^s\}$ with time resolution or scale factor *s* (*s* is an integer).

2 $$\begin{aligned} y_j^s=\frac{1}{s}\sum _{i=(j-1)s+1}^{js} x_i, ~~~~~~~~1\le j\le \frac{N}{s}. \end{aligned}$$

**Fig. 1:**
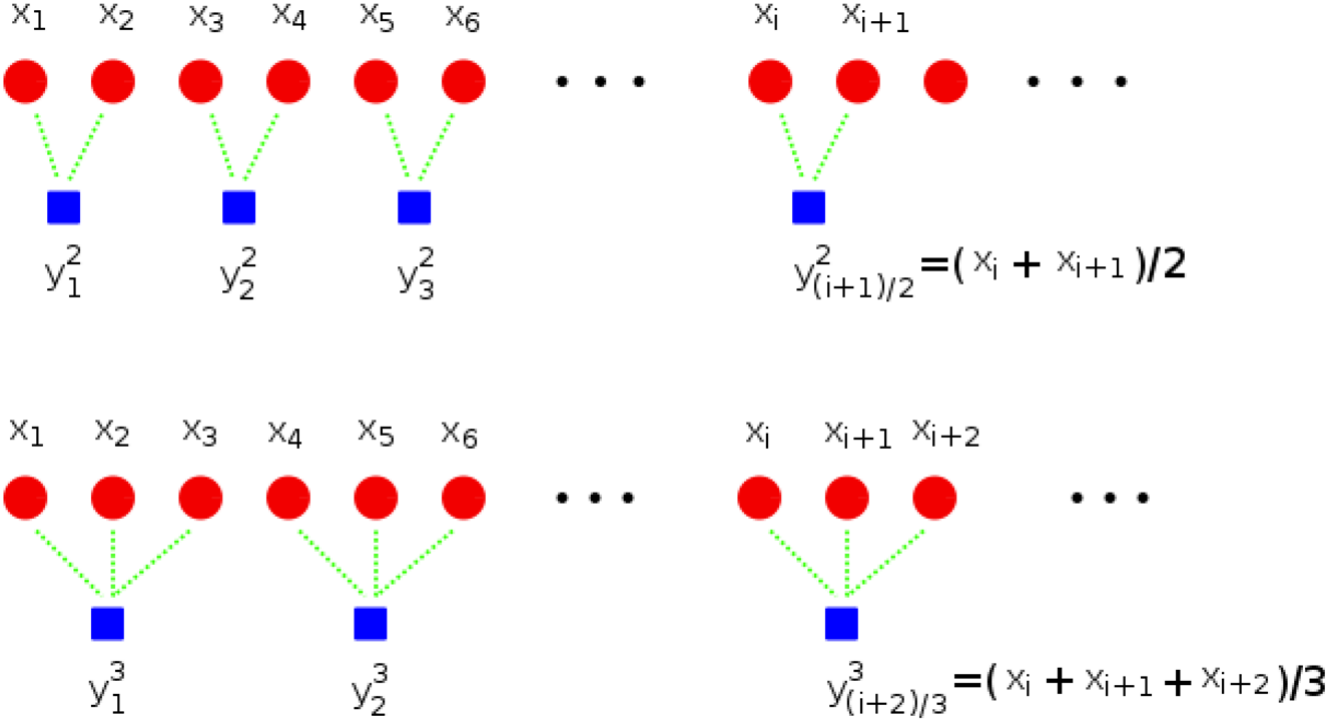
Calculation of multi-scale entropy for a coarse-grained time series with scale factors $s=2$ and $s=3$.

Each coarse-grained time series at scale factor *s* is obtained by averaging the samples of the original time series within a non-overlapping window of size *s*. The calculation of the coarse-grained time series is illustrated in Fig. 1. The length of each coarse-grained time series is *N*/*s*. The next step is to calculate the entropy for each coarse-grained time series.

#### Refined multi-scale entropy

Authors of research reported in^84^ show that calculation of multi-scale entropy is equivalent to applying a low-pass FIR filter to the signal and then performing sub-sampling. However, the authors argue that the frequency response of this FIR filter is feeble. Such a filter is characterized by a slow roll-off of the main lobe, a large transition band, and important side lobes in the stop band. This poor frequency response results in aliasing, generating spurious oscillations that affect entropy estimation. The authors propose redefining the multi-scale entropy estimation by first filtering the signal using a Butterworth low-pass filter in both the forward and backward directions to attain zero-phase filtering. The coarse-grained time series is obtained from the filtered signal, and entropy features are then estimated.

### Composite multi-scale entropy

One key limitation of multi-scale entropy is the increase in the variance of the entropy estimator as the length of the coarse-grained time series decreases. This limitation is an essential factor to consider when dealing with multi-scale entropy. The length of an AE time series sampled at high frequency is not an issue. However, using a long time series window for feature estimation significantly impacts the computational cost of estimating some non-linear entropy measures. Consequently, using non-linear information entropy measures with large signal windows is convenient. The Composite Multi-scale Entropy (CMSE) algorithm was developed to reduce the variance of the entropy estimation at large scales^58^.

**Fig. 2:**
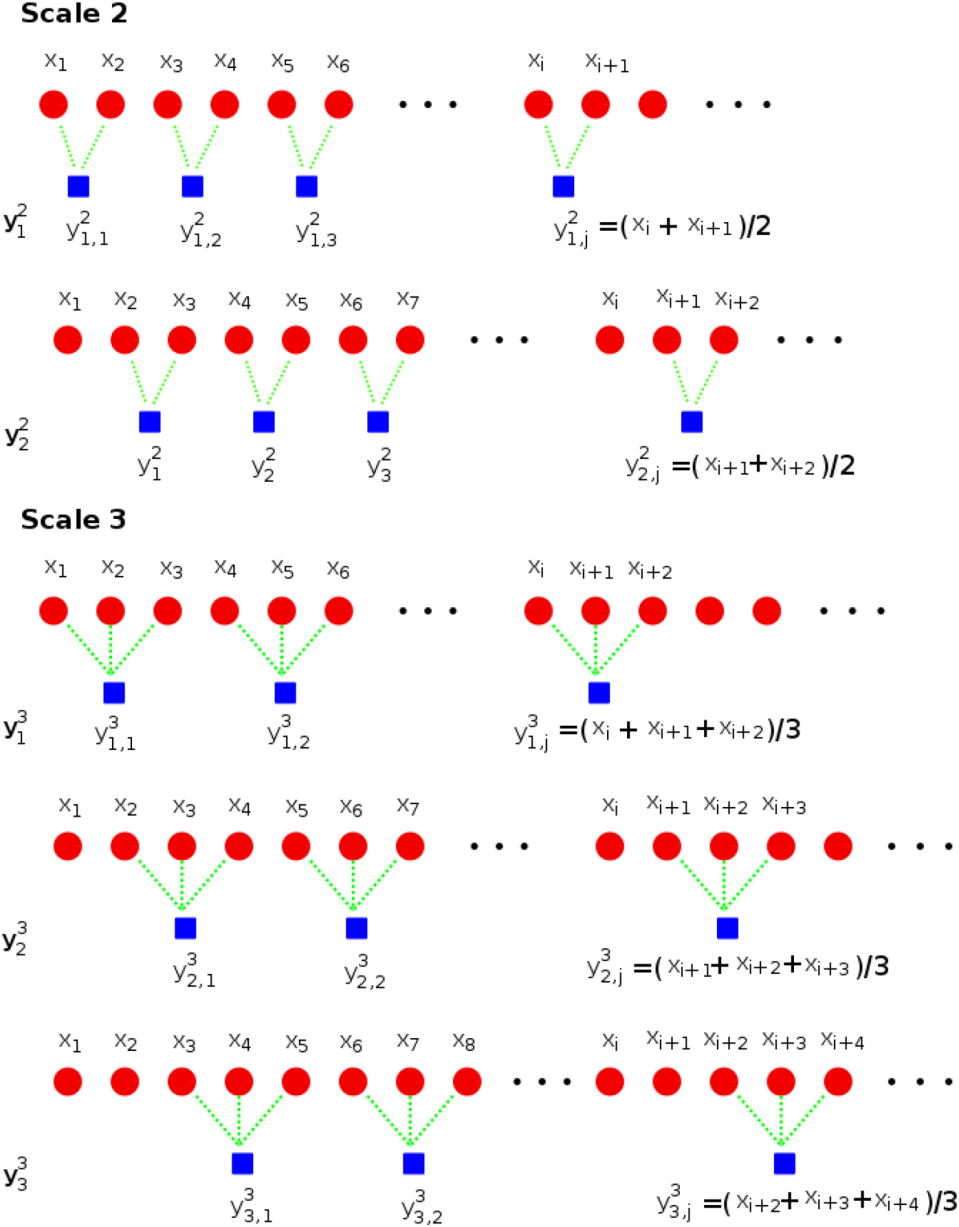
Calculation of the composite multi-scale entropy for scale factors $s=2$ and $s=3$.

The calculation of the composite multi-scale entropy is illustrated in Fig. 2. In this case, the calculation is shown for scale factors $s=2$ and $s=3$. The *kth* coarse-grained time series for scale *s* is denoted as $\textbf{y}_k^s=\{y_{k,1}^s, y_{k,2}^s,..., y_{k,p}^s\}$, with elements defined as:

3 $$\begin{aligned} y_{k,j}^s=\frac{1}{s} \sum _{i=(j-1)s+k}^{js+k-1} x_i,~~~~~~~1 \le j \le \frac{N}{s}, ~~~~~1\le k\le s \end{aligned}$$

When considering scale factors $s=2$ and $s=3$ we can see in Fig. 2 that in the case of $s=2$ there are two possible coarse-grained time series (denoted as $\textbf{y}_1^2, \textbf{y}_2^2$) that can be calculated and in the case of $s=3$ there are three possible coarse-grained time series (denoted as $\textbf{y}_1^3, \textbf{y}_2^3, \textbf{y}_3^3$). There are *p* coarse-grained time series for each scale factor *s*.

The entropy feature is estimated in this case for each coarse-grained time series at each scale factor, and these features are then averaged^83^ for each scale. For instance, if the calculated feature is the sample entropy, the calculation for the scale factor *s* is performed as:

4 $$\begin{aligned} CMSE(\textbf{x},s,m,r)=\frac{1}{s}\sum _{p=1}^s SampEn(\textbf{y}_p^s,m,r) \end{aligned}$$

A variant of the CMSE method, known as the refined composite multi-scale entropy (RCME), was reported in^85^. The variant, rather than averaging the sample entropy of each coarse-grained time series, averages the matched vectors used in the sample entropy calculation across all coarse-grained time series.

### Hierarchical multi-scale entropy

The hierarchical multi-scale entropy was developed to improve entropy estimation by overcoming the limitations of the multi-scale estimation algorithm. In particular, the hierarchical multi-scale entropy (HME)^61^ considers a lower frequency component obtained by averaging the signal components in the previous scale. In addition, the HME algorithm considers a higher frequency component obtained by calculating the difference between two consecutive scales. The HME approach can be implemented to estimate any non-linear entropy-based or statistical feature.

Given a time series of length *N*, denoted $\textbf{x}=[x_1, x_2, x_3,..., x_N]$, it is feasible to define an averaging operator $Q_0$ defined for $\textbf{x}$ as:

5 $$\begin{aligned} Q_0(x)=\frac{x_{2j}+x_{2j+1}}{2}, ~~~~~for ~~~~j=0,1,2,3, ...,2^{n-1}. \end{aligned}$$

where $N=2^n$, with *n* a positive integer. The time series $Q_0(x)$ is the low frequency of the original time series $\textbf{x}$, and its length is $2^{n-1}$ at scale 2. Similarly, an additional operator $Q_1$ can be defined as:

6 $$\begin{aligned} Q_1(x)=\frac{x_{2j}-x_{2j+1}}{2}, ~~~~~for ~~~~j=0,1,2,3, ...,2^{n-1} . \end{aligned}$$

The time series $Q_1(x)$ is the high frequency of $\textbf{x}$, and its length is $2^{n-1}$ at scale 2. The original time series $\textbf{x}$ can be reconstructed based on components $Q_0(x)$ and $Q_1(x)$ as:

7 $$\begin{aligned} \textbf{x}=\{(Q_0(x)_j+Q_1(x)_j),(Q_0(x)_j-Q_1(x)_j)\},~~~j=0,1,2,...,2^{n-1} \end{aligned}$$

When the integer $p \in \{0,1\}$, the operator *Q* can be represented in matrix form as:

8 $$\begin{aligned} Q_p(x)=\left[ \begin{array}{rrrrrrr} \frac{1}{2}& \left( \frac{-1}{2}\right) ^p& 0& 0& ...& 0& 0\\ 0& 0& \frac{1}{2}& \left( \frac{-1}{2}\right) ^p& ...& 0& 0\\ .& .& .& .& ...& .& .\\ 0& 0& 0& 0& ...& \frac{1}{2}& \left( \frac{-1}{2}\right) ^p\\ \end{array} \right] _{2^{n-1}\times 2^n}. \end{aligned}$$

The hierarchical representation of the signal using the operator $Q_p$ to attain the multi-scale analysis requires the repeated application of such operators. A node defined by the integer *u* in the hierarchical representation of the signal at the layer *k* can be represented as:

9 $$\begin{aligned} u=\sum _{m=1}^k \epsilon _m2^{k-m}, \end{aligned}$$

where $[\epsilon _1,\epsilon _2,...,\epsilon _k]$ is the binary representation of the integer *u* and $\epsilon _m \in \{0,1\}$. By designating in Equation (8), $p=\epsilon _m$, the hierarchical components of the original time series $\textbf{x}$ can be denoted as:

10 $$\begin{aligned} \textbf{x}_{k,u}=Q_{\epsilon _n}\cdot Q_{\epsilon _{n-1}}\cdot ... \cdot Q_{\epsilon _1} \cdot \textbf{x} \end{aligned}$$

**Fig. 3:**
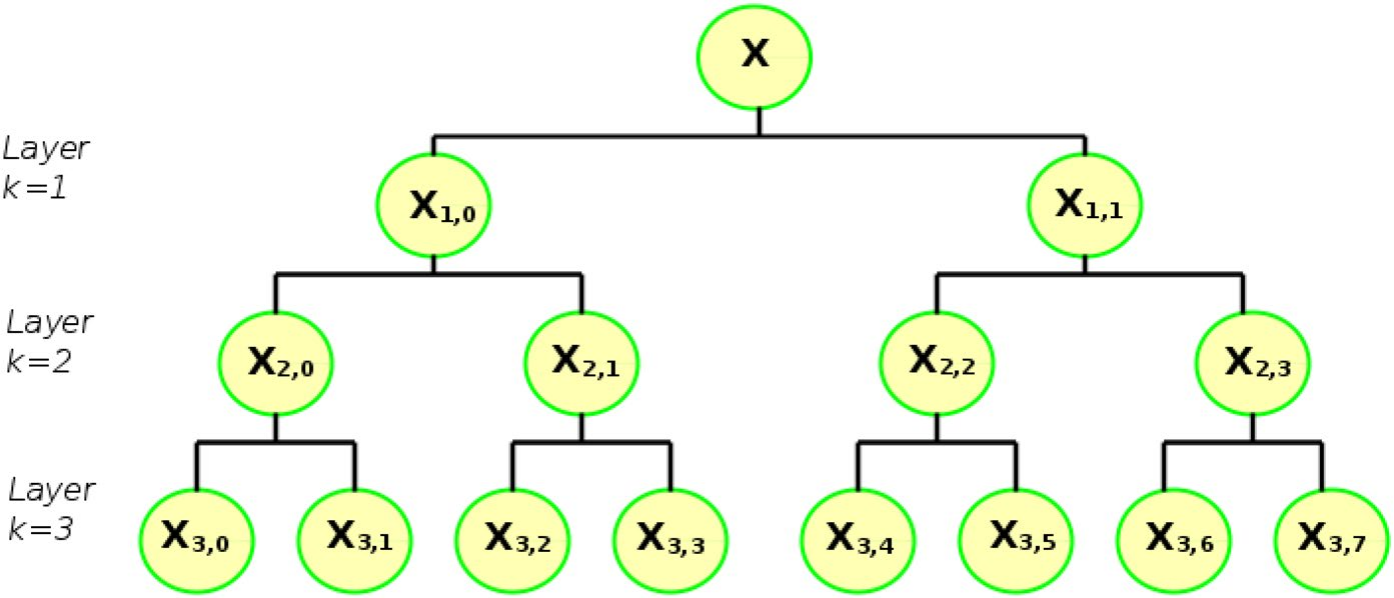
Graphical hierarchical decomposition with three hierarchical layers for calculation of the hierarchical multi-scale entropy.

$\textbf{x}_{k,u}$ represents the hierarchical decomposition of signal $\textbf{x}$ in multi-scale. The component $\textbf{x}_{k,0}$ with $k=1,2,...,N$ is equivalent to the multi-scale decomposition of the time series $\textbf{x}$ at scale $2^k$. A graphical representation of the hierarchical decomposition of signal $\textbf{x}$ with three layers is shown in Fig. 3.

### Composite hierarchical multi-scale entropy

A variant of the hierarchical multi-scale entropy is the composite hierarchical multi-scale entropy reported in^59^ for fault diagnosis in a planetary gearbox. The method represents an improvement of the multi-scale entropy estimation approach. Firstly, the first-order coarse-grained procedure is replaced by the hierarchical decomposition, which has proved to be an efficient method for fault detection^60,61^. Secondly, the approach incorporates the composite multi-scale entropy estimation approach to reduce the variance of the entropy estimation as the window length varies during the multi-scale approach^58^. The CHMSE approach performs the hierarchical decomposition of the signal. Then, the composite approach is applied to each node in a hierarchical layer to estimate the entropy features, and the mean value is calculated. The Algorithm 1 describes the CHMSE method.

**Algorithm 1:**
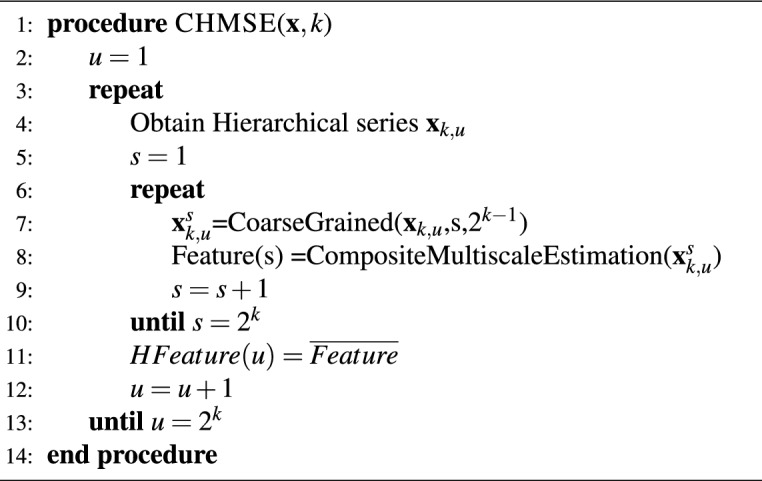
Composite Hierarchical Multi-scale Entropy

### Interpretability in fault diagnosis of rotating machinery

The transparency and interpretability of machine learning models have become increasingly critical in fault diagnosis of rotating machinery, where automated systems must provide not only accurate predictions but also understandable reasoning that practitioners can trust and act upon. In this context, two complementary approaches to achieving interpretability have emerged: intrinsic interpretability and post-hoc explainability^86^. Intrinsic interpretability refers to the inherent transparency of a model built directly into its structure during design and training, such as decision trees, linear regression models, or generalized additive models, which naturally encode their decision-making mechanisms through interpretable parameters and rules. Post-hoc explainability, conversely, applies explanation techniques retrospectively to complex “black-box” models–such as deep neural networks or random forests–that have already been trained, using model-agnostic methods like SHAP (SHapley Additive exPlanations) or LIME (Local Interpretable Model-agnostic Explanations) to approximate and communicate their internal decision logic^87^. In rotating machinery diagnostics, the choice between these approaches presents a trade-off between model interpretability and predictive accuracy. In contrast, intrinsically interpretable models offer transparency. Still, they may sacrifice performance when dealing with complex, multi-scale fault signatures. Post-hoc methods enable the deployment of high-performance models while providing transparency through algorithmic explanations^88^.

Complementary to the intrinsic-versus-post-hoc distinction, interpretability in fault diagnosis encompasses both global and local levels of explanation, providing different types of insights essential for industrial reliability and maintenance decision-making^89^. Global interpretability describes the overall influence of features on model behavior across the entire population of observations, revealing which physical parameters and signal characteristics the model considers universally important for fault classification. Local interpretability, by contrast, explains the model’s prediction for specific individual samples or instances, identifying which features and values contributed most strongly to a particular diagnostic decision. In practical rotating machinery monitoring, both scales of interpretation serve complementary functions: global interpretability guides feature engineering, sensor selection, and algorithm development by identifying universal fault signatures, while local interpretability supports real-time diagnosis by providing operators and maintenance engineers with instance-specific justifications that they can verify against physical understanding of the fault mechanism^90^. Recent applications of SHAP in bearing and gearbox diagnostics demonstrate how post-hoc, local-global interpretability analysis can simultaneously reveal which features drive overall classification performance while exposing the specific feature contributions underlying individual predictions, thereby bridging the gap between machine learning model transparency and domain knowledge^91^. Based on this theoretical foundation and the identified research gaps, we designed a comprehensive experimental study to systematically evaluate these entropy-based approaches on acoustic emission signals from gearbox faults.

## Experiment platform

### Acoustic emission signal acquisition

**Fig. 4:**
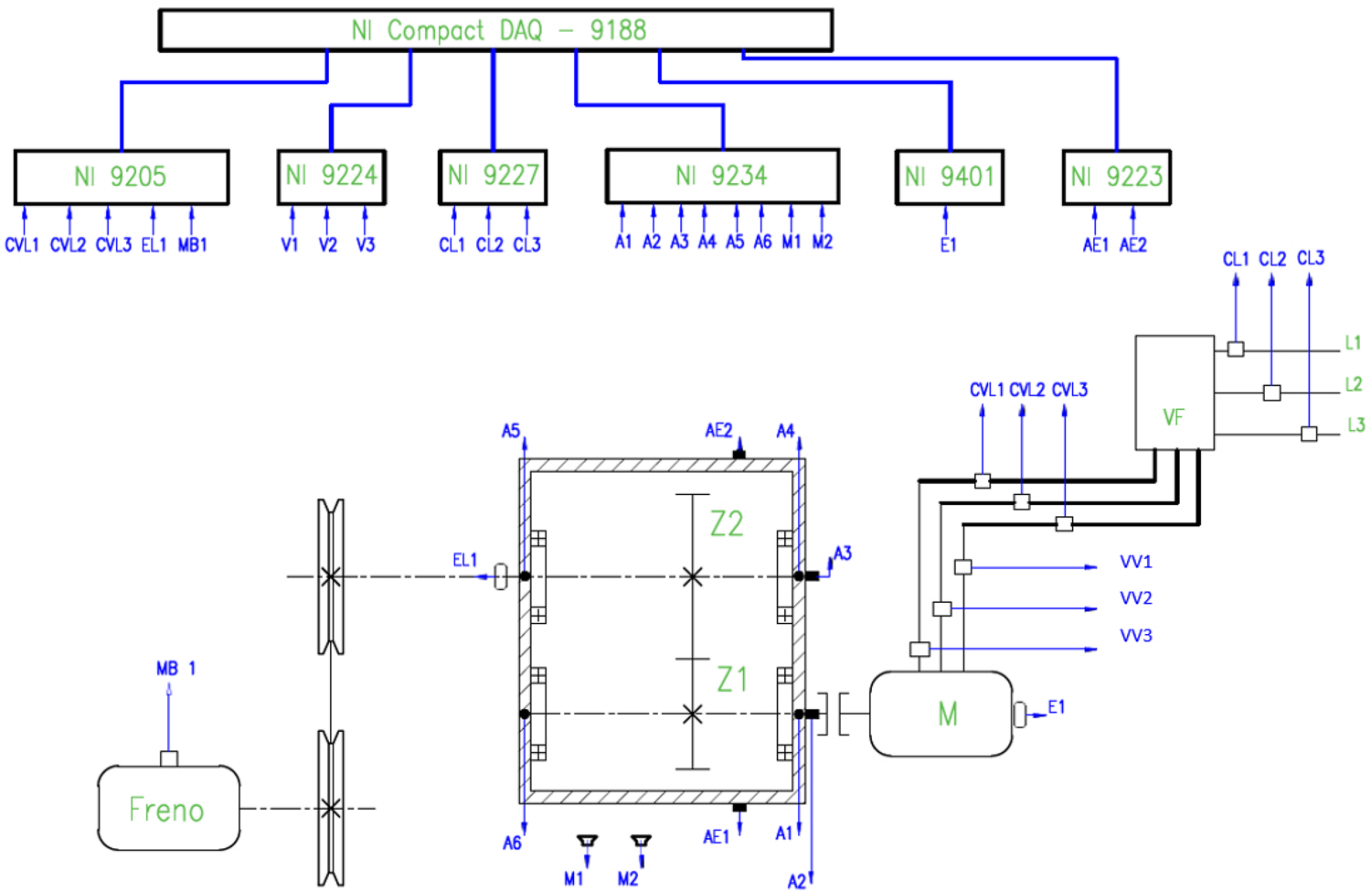
An experimental test rig for acquiring acoustic emission signals from a spur gearbox aimed at investigating the fault severity classification. The laboratory is located at the Salesian Polytechnic University, Cuenca, Ecuador.

The acoustic emission signal was acquired using an acoustic emission sensor (AE1) model Physical Acoustics WD mounted laterally in the gearbox. The spur gearbox was connected to the 2-hp motor through a shaft. The gearbox’s output shaft was connected to a magnetic brake through a belt to simulate different loads. Two spur gears, denoted Z1 and Z2, were inside the gearbox. The number of teeth was 32 for Z1 and 48 for Z2, with a modulus of 2.25 and an impact angle of $20^{\circ }$. The acoustic emission signal was amplified and sampled at 1 MS/s, and the collected data was transmitted via Ethernet to a laptop computer. The diagram showing the experimental test rig is presented in Fig. 4.

Four types of faults, each with nine fault severity levels, were implemented for acoustic emission signal acquisition. The condition denoted P1 corresponds to the healthy gearbox. The rest of the P2 to P9 conditions correspond to eight fault severity levels. The artificial creation of the pitting faults was attained using Electrical Discharge Machining (EDM). The implemented faults were pitting, broken teeth, cracks, and scuffing. The detailed description of the test rig is presented in^92^.

### Dataset acquisition with acoustic emission signals

Three loads were configured in the brake, and three constant speeds of 6, 12, and 18 Hz were programmed. Considering each combination of load and speed for each healthy condition, the signal was recorded for 3 seconds and sampled at 1 MHz. This process was repeated 15 times. In total, the dataset included 1215 signal samples. The details concerning each fault severity level and signal measurement are presented in^92^.

## Methodology for fault extraction and classification

**Fig. 5:**
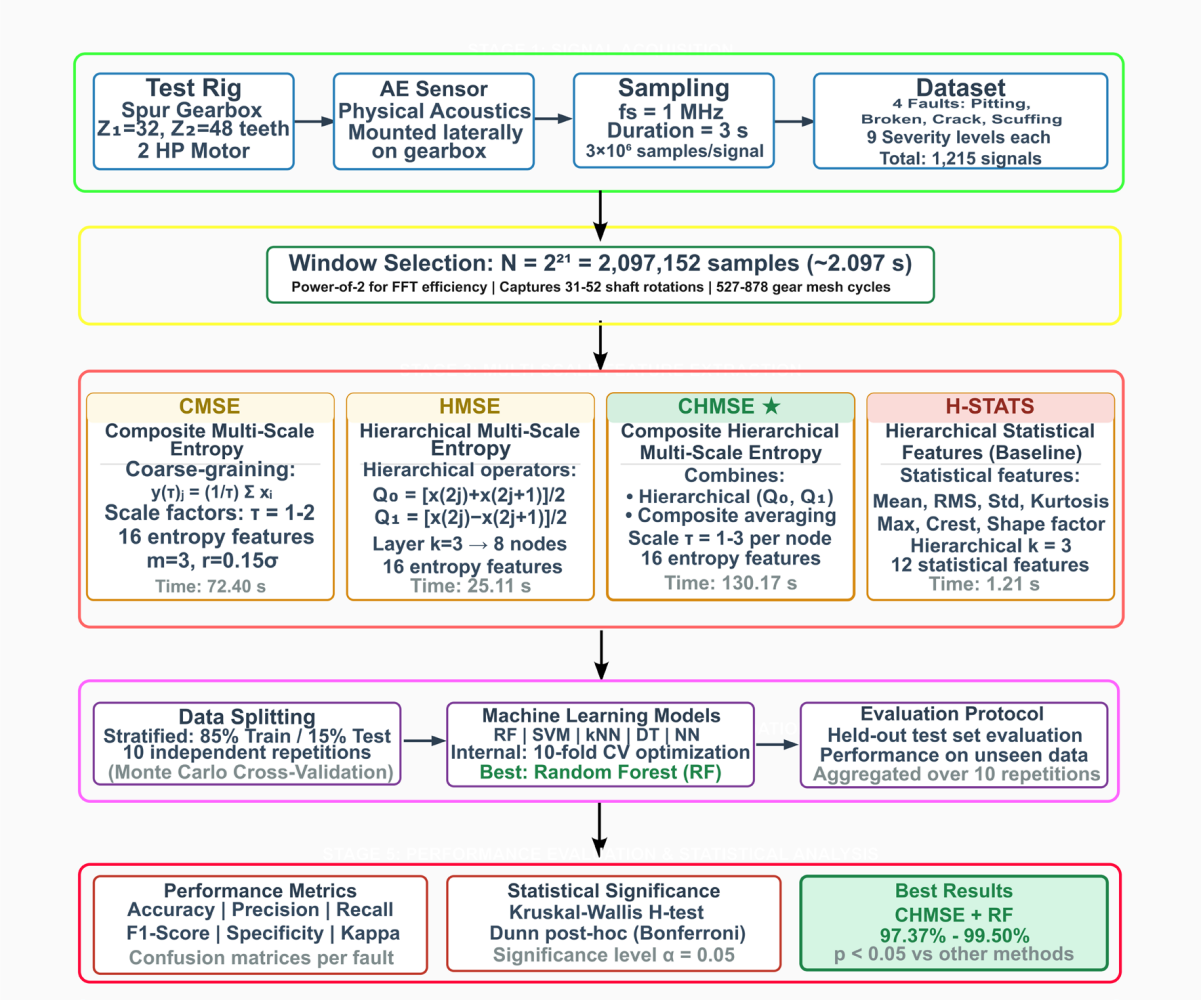
Complete methodology flowchart for multi-scale entropy analysis of acoustic emission signals for gearbox fault severity classification. The framework consists of five stages: (1) signal acquisition from the experimental test rig sampled at 1 MHz; (2) preprocessing with window selection ($N=2^{21}$ samples); (3) parallel multi-scale feature extraction using CMSE ($s=1$–2), HMSE ($k=3$, operators $Q_0$ and $Q_1$), CHMSE, and hierarchical statistical features; (4) classification using five machine learning models with stratified validation, and (5) performance evaluation with statistical significance testing.

This research pursued two primary objectives: (1) to systematically compare the performance of multi-scale entropy feature extraction methods (CMSE, HMSE, CHMSE), and (2) to identify a computationally efficient feature set achieving high classification accuracy across fault severity levels. In addition, a selection of non-linear features that should be accurate for severity classification and computationally efficient will be performed. A methodology for evaluating and comparing the performance of the multi-scale extraction in combination with non-linear information features is presented in Fig. 5. The composite multi-scale information entropy, the hierarchical multi-scale information entropy, and the composite hierarchical multi-scale information entropy were compared with the hierarchical multi-scale statistical features in terms of their accuracy for classifying fault severity across four fault types.

The high-frequency sampling rate of the AE signal poses severe challenges for feature extraction methods. In particular, in the non-linear features, whose calculation is centered on distance estimation between vectors of the phase-space reconstruction in equation (1), the computational cost increases heavily with the size *N* of the time series. This fact limits the value of *N*.

To avoid this limitation, we have selected an entropy-based feature set that does not require vector distance calculations and is both computationally efficient and accurate for fault severity classification. Table 1 presents the selected feature set. The selected entropy features are mainly based on information entropy, which is computationally efficient and can be applied to large signal windows without significant increments in computation time. Information-based entropy features include permutation entropy and information entropy. The wavelet packet transform is used for processing the signal, and several entropy measures are estimated: the norm entropy, the log-energy entropy, the threshold entropy, and the “sure” entropy.

**Table 1:** Selected non-linear information entropy feature set. The features are composed of two groups. The first group, with features 1–8, consists of permutation entropy and wavelet packet-based features. The second group with features 9–16 corresponds to spectral entropy-based features.

**Number**	**Feature**	**Equation/Reference**	**Parameters**
1	Permutation entropy	^71^	m=3
2	Rényi entropy	^75^	$q_R=0.5$
3	Tsallis entropy	^75^	$q_T=1.5E-5$
4	Information entropy	^74^	
5	Log energy entropy	^76^	
6	Norm entropy	^76^	$p_N=1.8$
7	Threshold entropy	^76^	$p_{Thres}=3.5std(x)$
8	The “sure” entropy	^76^	$p_{S}=3.5std(x)$
9	Mean value of spectral entropy$\textbf{S}$	mean($\textbf{S}$)	$\textbf{S}$=Instantaneous spectral entropy
10	Standard deviation of$\textbf{S}$	std($\textbf{S}$)	
11	RMS value of$\textbf{S}$	rms($\textbf{S}$)	
12	Shape factor of$\textbf{S}$	$\frac{rms(\textbf{S})}{\frac{1}{N}\sum _i \left| s_i \right| }$	
13	Maximum to rms ratio	max($\textbf{S}$)/rms($\textbf{S}$)	
14	Median value of$\textbf{S}$	median($\textbf{S}$)	
15	Skewness of$\textbf{S}$	skewness($\textbf{S}$)	
16	Kurtosis of$\textbf{S}$	kurtosis($\textbf{S}$)	

Additionally, the Rényi and Tsallis entropies are estimated for the signal. Spectral entropy is estimated from the time-frequency representation of the acoustic emission signal. Then, a set of features is extracted corresponding to the mean value of spectral entropy, the standard deviation of spectral entropy, the root mean square value of spectral entropy, the shape factor of spectral entropy, the ratio between the maximum to the RMS value of spectral entropy, the median value of the spectral entropy, the skewness of spectral entropy and the kurtosis of spectral entropy. The selected feature set was used to compare multi-scale entropy estimation algorithms across four fault severity types: pitting, broken teeth, root cracks, and scuffing. In addition, a comparison to a standard statistical feature set was performed. Table 2 presents the statistical feature set. A set of 12 statistical features is extracted to compare with the multi-scale entropy features set. The performance of each feature set was evaluated using several classical machine learning algorithms and a severity classification.

### Features extraction procedure

#### Information entropy feature extraction

The non-linear information entropy features are computationally efficient, and the analysis signal window length can be extended without a significant impact on computation time. The extracted features are presented in Table 1. In this research, we have selected the highest-performing multi-scale algorithms, namely composite multi-scale entropy (CMSE), hierarchical multi-scale entropy (HMSE), and composite hierarchical multi-scale entropy (CHMSE). The $k=3$ layer was used to perform the hierarchical multi-scale analysis. Concerning the composite hierarchical multi-scale entropy, only two scales were considered. Each acquired acoustic emission signal consisted of 3 seconds of continuous data sampled at 1 MS/s, yielding 3,000,000 samples per acquisition. For entropy-based feature extraction, a single analysis window of $2^{21} = 2,097,152$ samples (approximately 2.097 seconds) was extracted from the initial portion of each signal, starting from the first sample.

**Table 2:** Statistical feature set (STATS).

**Number**	**Feature**	**Equation**
1	Mean ($\mu$)	$\frac{1}{N} \sum _{i=1}^N x_i$
2	Root Mean Square (RMS)	$\sqrt{ \frac{1}{N} \sum _{i=1}^N \left( x_i \right) ^2 }$
3	Standard deviation ($\sigma$)	$\sqrt{ \frac{1}{N} \sum _{i=1}^N \left( x_i -\mu \right) ^2 }$
4	Kurtosis	$\frac{ N \sum _{i=1}^N \left( x_i -\mu \right) ^4 }{ \left[ \sum _{i=1}^N \left( x_i -\mu \right) ^2 \right] ^2 }$
5	Maximum value	$max(\textbf{x}_n)$
6	Crest factor	$\frac{ max(\textbf{x}_n)}{rms(\textbf{x}_n) }$
7	Rectified mean value	$\frac{1}{N} \sum _{i=1}^N \left| x_i \right|$
8	Shape factor	$\frac{rms(\textbf{ x}_n)}{\frac{1}{N} \sum _{i=1}^N \left| x_i \right| }$
9	Impulse factor	$\frac{max(\textbf{ x}_n)}{\frac{1}{N} \sum _{i=1}^N \left| x_i \right| }$
10	Variance	$\frac{1}{N} \sum _{i=1}^N \left( x_i -\mu \right) ^2$
11	Min value	$min(\textbf{x}_n)$
12	Skewness	$\frac{ N \sum _{i=1}^N \left( x_i -\mu \right) ^3 }{ \sigma ^3 }$

#### Feature extraction of statistical features

Twelve statistical features were extracted from the acoustic emission signals. Such features have been used in our laboratory to validate several approaches of fault classification in rotating machinery^93^. This feature set was selected using feature selection methods from a more extensive set of statistical features. The statistical features are listed in Table 2. The statistical features used for comparison were extracted using the hierarchical multi-scale entropy. The window length was kept identical to the case of non-linear information entropy estimation.

### Faults classification based on the extracted features

Several machine learning models were considered for fault classification: random forests (RF)^94^, support vector machines (SVM)^95^, k-Nearest Neighbors classifiers (kNN)^96^, decision trees (DT)^97^, and neural networks (NN)^98^.

The validation protocol employed repeated Monte Carlo cross-validation: (1) The dataset was randomly split 10 times into training (85%, n=1,033) and test sets (15%, n=182), stratified to maintain class distribution. (2) For each split, the training set was subjected to 10-fold stratified cross-validation for model optimization. (3) Final performance metrics^99,100^ were computed on the held-out test set and aggregated across all 10 repetitions to provide robust performance estimates with confidence intervals.

#### Statistical significance testing

To rigorously assess whether observed performance differences among multi-scale entropy feature extraction methods (CMSE, HMSE, CHMSE) and hierarchical statistical features are statistically significant, a comprehensive nonparametric statistical analysis was conducted on test-set accuracy results from 10 independent validation repetitions.

The statistical analysis addresses the following hypotheses:

1. **Null hypothesis** ($H_0$): All four feature extraction methods yield equivalent classification accuracy
2. **Alternative hypothesis** ($H_1$): At least one method produces significantly different accuracy compared to others.

Given that each method was evaluated across 10 independent repetitions (n = 10 per group) and that the sample size was modest, without prior assumptions about data distribution normality, the Kruskal-Wallis test was selected as the primary statistical test. The Kruskal-Wallis test is a nonparametric alternative to one-way ANOVA that compares medians across multiple independent groups without requiring normality assumptions, making it appropriate for machine learning performance metrics that may exhibit non-Gaussian distributions.

When the Kruskal-Wallis test indicated significant differences among groups ($p < 0.05$), post-hoc pairwise comparisons were conducted using MATLAB’s multcompare function, which implements the Tukey-Kramer procedure for multiple comparisons. This method controls the family-wise error rate (FWER) when performing multiple pairwise tests, adjusting critical values to account for the increased probability of Type I errors inherent in multiple comparisons. For k = 4 groups, this procedure evaluates six pairwise comparisons.

All statistical tests were conducted at a significance level of $\alpha = 0.05$, corresponding to 95% confidence intervals. A *p*-value below 0.05 indicates sufficient evidence to reject the null hypothesis of no difference between methods.

## Results

The complete experimental dataset comprised 1,215 acoustic emission signal samples collected from a spur gearbox under controlled operating conditions. The dataset encompassed four fault mechanisms (pitting, broken teeth, root cracks, and scuffing), each represented by nine severity levels (P1–P9), acquired at three load configurations and three rotational speeds (6, 12, and 18 Hz). Each signal consisted of 3 seconds of continuous data sampled at 1 MHz, yielding 3,000,000 samples per acquisition.

**Fig. 6:**
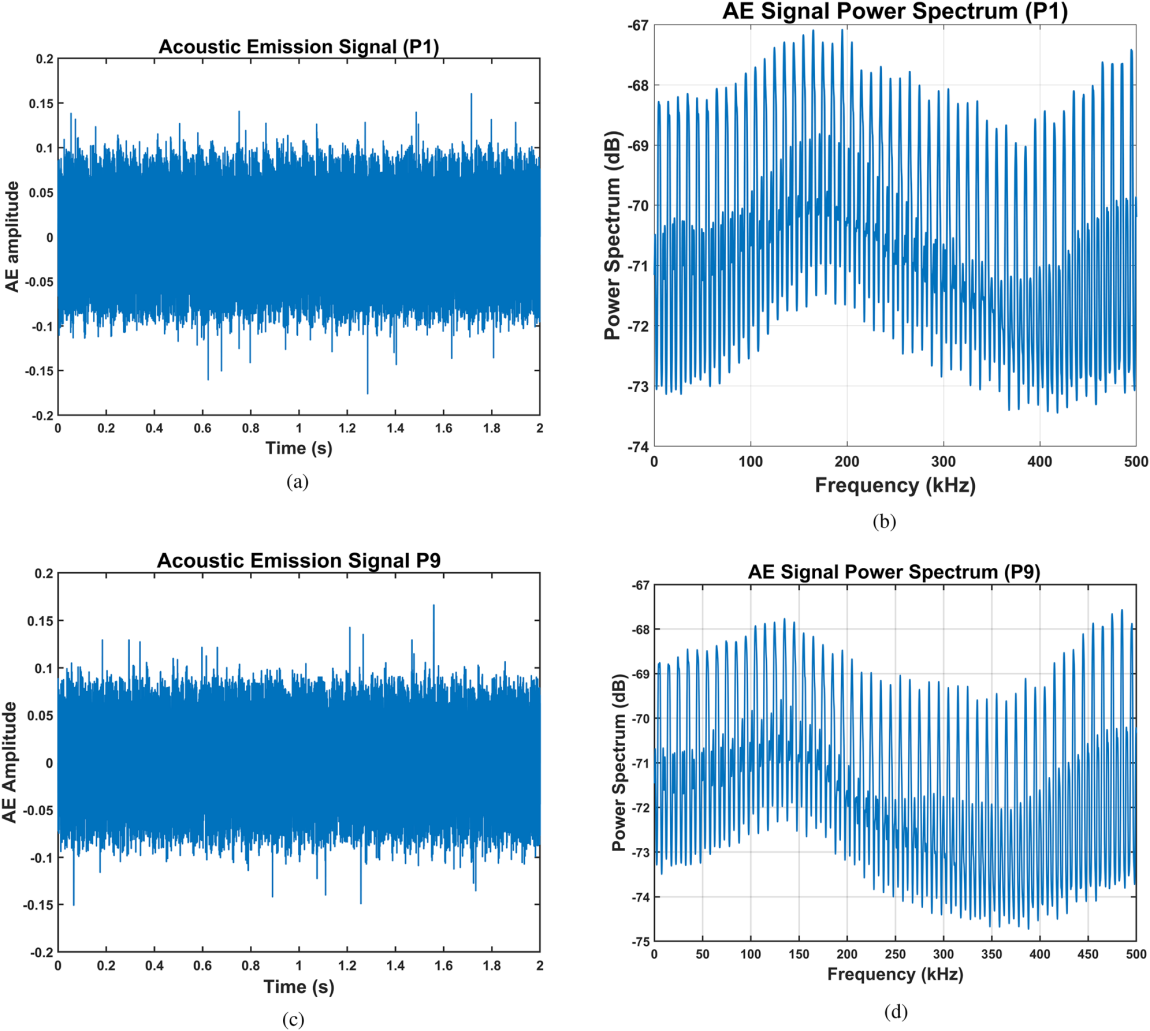
AE for the spur gearbox. (**a**) Time-domain signal representation for P1 pitting severity, (**b**) power spectrum for P1 pitting severity, (**c**) Time-domain signal representation for P9 pitting severity, and (**d**) power spectrum for P9 pitting severity.

Figure 6 presents representative time-domain acoustic emission signals and their corresponding power spectra for pitting faults at two severity extremes: the healthy baseline condition (P1, Fig. 6a,b) and the most advanced damage state (P9, Fig. 6c,d). Visual inspection reveals minimal discernible differences between the time-domain waveforms (Fig. 6a,c) and their frequency spectra (Fig. 6b,d). Both the healthy and severely damaged gears generate broadband acoustic emissions across the 1 MHz sampling bandwidth, with no distinctive fault signatures evident in conventional time-domain or frequency-domain representations. This absence of visually distinguishable features in standard signal analyses underscores the need for advanced, non-linear, entropy-based feature-extraction methods capable of revealing subtle changes in signal complexity associated with progressive fault development.

### Comparison between statistical and non-linear information features

**Table 3:** Hierarchical multi-scale performance comparison: random forest classification accuracy and related metrics for statistical vs. non-linear information entropy features.

**Features type**	**Model**	**Accuracy**	**Sensitivity**	**Specificity**	**Precision**	**FPR**	**Kappa**
Statistical	Pitting	97.72	97.76	99.72	97.67	0.28	88.47
	Broken	97.67	97.72	99.71	97.73	0.29	88.22
	Crack	96.73	96.83	99.59	96.76	0.41	83.46
	Scuffing	95.40	95.39	99.43	95.35	0.57	76.69
Non-linear information entropy	Pitting	98.02	98.10	99.75	98.01	0.25	89.98
	Broken	99.01	99.01	99.88	99.06	0.12	94.99
	Crack	**99.26**	**99.28**	**99.91**	**99.27**	**0.09**	**96.24**
	Scuffing	97.33	97.37	99.67	97.37	0.33	86.47

The statistical features listed in Table 2 and the non-linear information entropy features in Table 1 were extracted from each fault type severity dataset using the hierarchical multi-scale approach. Each classical model was evaluated on the held-out test set using the extracted features. The best results were attained with the RF model in each fault type. Table 3 presents the random forests model evaluation results on the held-out test set. The highest accuracy attained with statistical features of the pitting fault severity dataset, 97.72%, is lower than that achieved with the information entropy features extracted with the hierarchical multi-scale method, 98.02%. The accuracy achieved with hierarchical multi-scale statistical features extracted from the broken tooth dataset was 97.67%, which is lower than that with non-linear information entropy (99.01%). Similarly, for the root crack fault severity, the average accuracy was 96.73%, while the non-linear information entropy attained 99.26%. Regarding scuffing fault severity, the accuracy with the statistical feature set was 95.40%, while the non-linear information entropy features achieved an average accuracy of 97.33%. In conclusion, when considering the hierarchical multi-scale feature extraction method, the non-linear information entropy feature set achieves higher accuracy across fault types.

#### Fault severity classification using composite hierarchical multi-scale features

The composite hierarchical multi-scale feature extraction method was applied to the AE pitting fault severity dataset, and evaluation results on the held-out test set for several machine learning models are presented in Table 4. The random forests model scored first with an accuracy of 97.87%, while the decision trees model scored second with a classification accuracy of 94.16%. The remaining metrics vary consistently with classification accuracy.

**Fig. 7:**
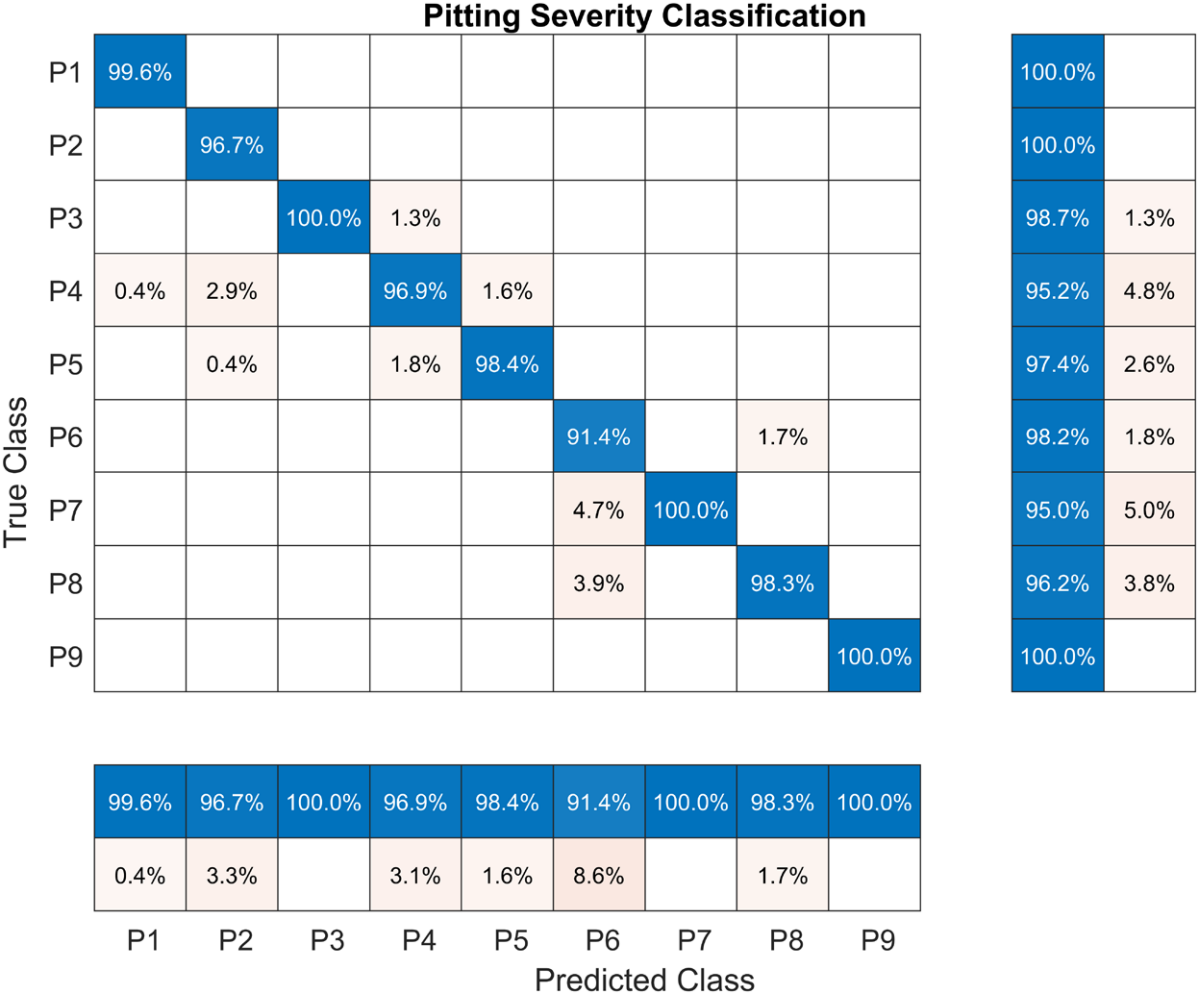
Confusion matrix for the RF model trained with CHMSE features extracted from the pitting fault severity dataset.

The confusion matrix for the RF model, evaluated on the held-out test set with non-linear information entropy features extracted from the pitting fault severity dataset, is shown in Fig. 7. The P7 class had the lowest TPR of 95.0%. Classes P1, P2, and P9 attained a value of 100%, which is the highest. Concerning the PPV, class P6 attained the lowest value of 91.4% while classes P3, P7, and P9 attained the highest value corresponding to 100%.

**Table 4:** Performance metrics for each of the machine learning models.The models were trained with the CHMSE features extracted from the pitting fault severity dataset.

**Model**	**Accuracy**	**Sensitivity**	**Specificity**	**Precision**	**FPR**	**Kappa**
RF	**97.87**	**97.85**	**99.73**	**97.92**	**0.27**	**89.22**
NN	91.83	92.01	98.98	91.93	1.02	58.65
SVM	93.51	93.81	99.19	93.60	0.81	67.17
kNN	82.08	82.00	97.76	81.92	2.24	9.28
DTrees	94.16	94.09	99.27	94.05	0.73	70.43

Results of the evaluation on the held-out test set concerning each of the machine learning models trained with features extracted from the broken tooth fault severity are presented in Table 5. Non-linear information-entropy features were extracted using the composite hierarchical approach. As in the other experiments, the random forest models achieved the best performance, with an accuracy of 99.70%, while the SVM model ranked second with 97.62%.

**Fig. 8:**
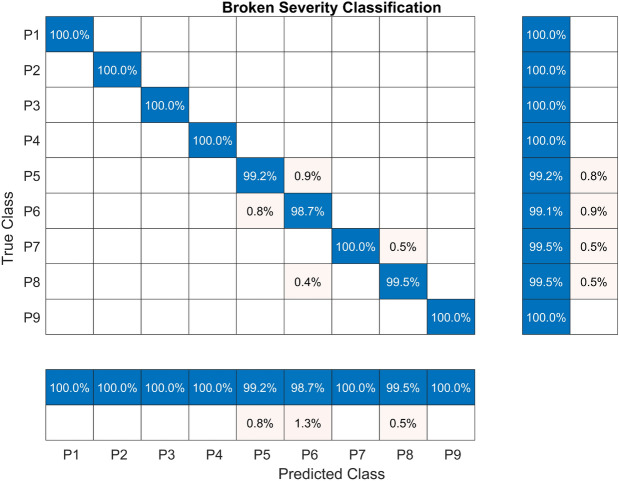
Confusion matrix for the RF model trained with CHMSE features extracted from the broken teeth fault severity dataset.

The confusion matrix for this experiment is presented in Fig. 8. The TPR results are excellent, with the lowest value of 99.1% attained by class P6. Classes P1-P4 and P9 attained the highest value of 100%. Similarly, the lowest value of PPV was 98.7%, attained by class P6, while classes P1-P4, P7, and P9 attained a value of 100.00 %, which is the highest.

**Table 5:** Performance metrics of the machine learning models.The models were trained with the CHMSE features extracted from the broken tooth fault severity dataset.

**Model**	**Accuracy**	**Sensitivity**	**Specificity**	**Precision**	**FPR**	**Kappa**
RF	**99.70**	**99.70**	**99.96**	**99.71**	**0.04**	**98.50**
NN	94.95	94.95	99.37	94.94	0.63	74.44
SVM	97.62	97.63	99.70	97.59	0.30	87.97
kNN	85.64	86.16	98.21	85.70	1.79	27.32
DTrees	93.56	93.71	99.20	93.58	0.80	67.42

**Fig. 9:**
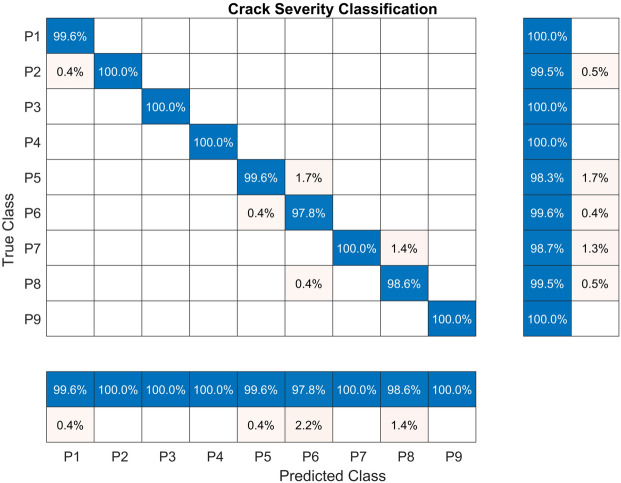
The confusion matrix for the RF model trained with the CHMSE features extracted from the root crack.

The results concerning the evaluation on the held-out test set of machine learning models trained with features extracted from the root crack fault severity are presented in Table 6. As with the rest of the experiments, the RF model scored first with an accuracy of 99.50%, while the SVM achieved 97.33%. The lowest accuracy was attained with the kNN model. The remaining metrics were consistent with the accuracy value. The confusion matrix for this experiment is presented in Fig. 9. The lowest True Positive Rate (TPR) of 98.3% was attained by class P5, while classes P1, P3, P4, and P9 attained the highest value of 100%. The lowest Predictive Positive Value (PPV) was achieved by class P6 with 97.8%, while classes P2-P4, P7, and P9 attained the highest PPV corresponding to 100%.

**Table 6:** Classification accuracy for each machine-learning model. The models were trained with the CHMSE non-linear entropy features extracted from the root crack severity fault.

**Model**	**Accuracy**	**Sensitivity**	**Specificity**	**Precision**	**FPR**	**Kappa**
RF	**99.50**	**99.51**	**99.94**	**99.51**	**0.06**	**97.49**
NN	95.94	95.95	99.49	95.95	0.51	79.45
SVM	97.33	97.29	99.67	97.36	0.33	86.47
kNN	87.18	87.27	98.40	87.11	1.60	35.09
DT	91.93	92.02	98.99	91.94	1.01	59.15

The results of the evaluation on the held-out test set of classical machine learning models are presented in Table 7. The models were trained with non-linear information entropy features extracted from the scuffing dataset. As in the previous experiment, the RF model achieved the best performance with an accuracy of 98.17%. At the same time, the second place was attained by the SVM model with 90.79%, which represents an accuracy lower than that of the RF model (7.38%). The kNN model attained the lowest accuracy in this experiment, at 82.33%.

**Fig. 10:**
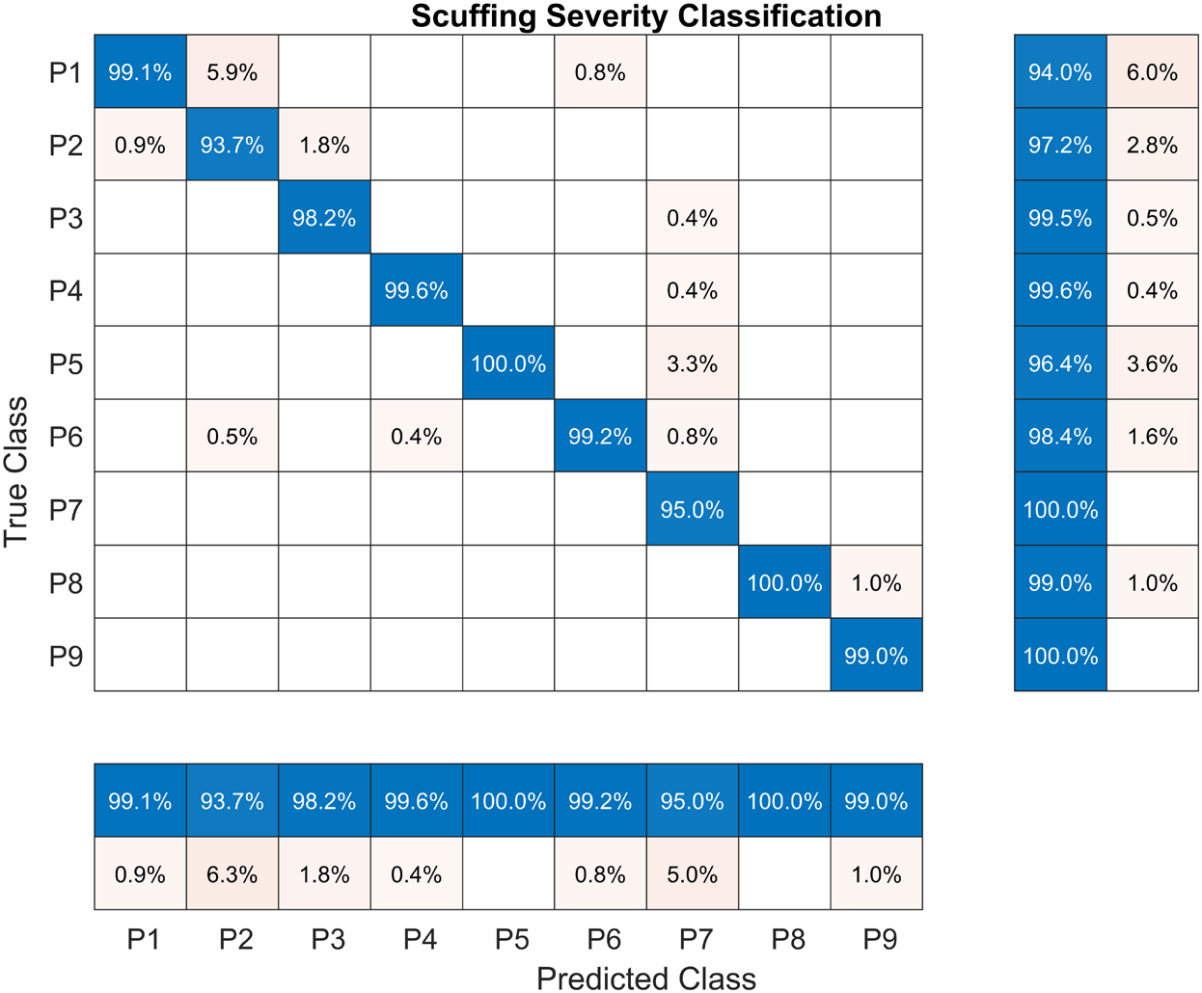
Confusion matrix for the RF model trained with the CHMSE features extracted from the scuffing fault severity dataset.

The confusion matrix for the experiment with the scuffing fault severity dataset is presented in Fig. 10. The TPR varies between 98.3 % for class P5 to 100% concerning the classes P1, P3, P4, and P9. The PPV of 97.8% is the lowest and was attained by class P6. The highest value of PPV was 100% attained by classes P2-P4, P7, and P9.

**Table 7:** Performance metrics for the machine learning models.The models were trained using CHMSE features extracted from the scuffing fault severity dataset.

**Model**	**Accuracy**	**Sensitivity**	**Specificity**	**Precision**	**FPR**	**Kappa**
RF	**98.17**	**98.23**	**99.77**	**98.20**	**0.23**	**90.73**
NN	88.07	88.12	98.51	88.09	1.49	39.60
SVM	90.79	90.80	98.85	90.76	1.15	53.38
kNN	82.33	82.37	97.80	82.07	2.20	10.53
DTrees	86.19	86.27	98.28	86.03	1.72	30.08

### Comparison between multi-scale feature extraction methods

A comparison between the best performance multi-scale feature extraction methods corresponding to the composite multi-scale entropy, the hierarchical multi-scale entropy, and the composite hierarchical multi-scale entropy was performed using the non-linear entropy features. In addition, we considered the hierarchical multi-scale method for extracting statistical features.

**Fig. 11:**
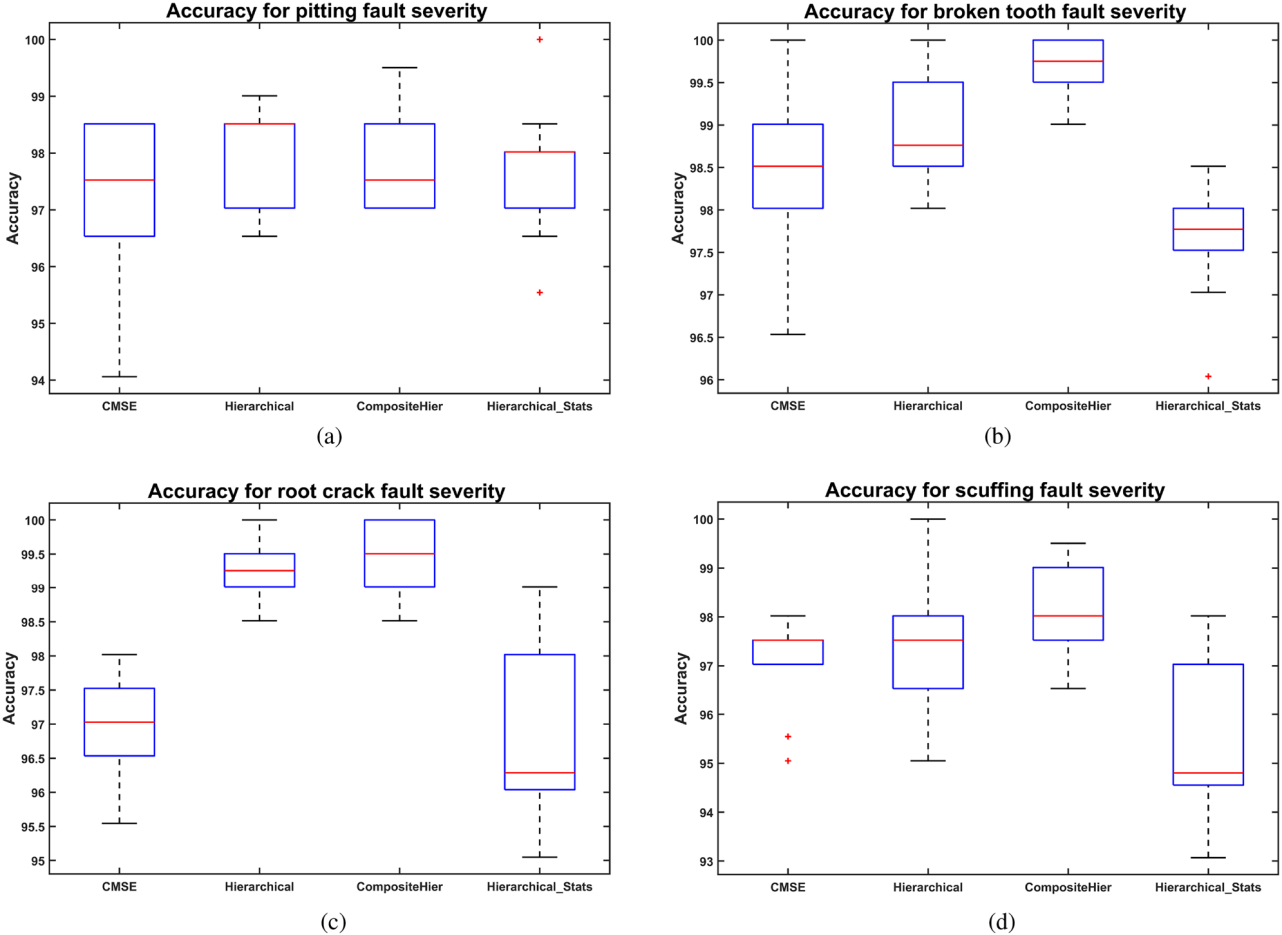
Comparison of performance obtained during the evaluation on the held-out test set of the RF model with features extracted using several multi-scale approaches. (**a**) Pitting fault severity dataset, (**b**) broken tooth fault severity dataset, (**c**) crack fault severity dataset, and (**d**) scuffing fault severity dataset.

A performance comparison obtained during evaluation on the held-out test set of the RF model with features extracted from the pitting fault severity dataset is shown in Fig. 11a. The composite, multi-scale, hierarchical, and composite hierarchical multi-scale methods are compared in terms of performance for extracting non-linear information entropy features. In addition, the comparison includes the performance of statistical features extracted using the hierarchical multi-scale approach. However, there are no statistically significant differences. The hierarchical and composite hierarchical multi-scale methods exhibit low variance and are unaffected by outliers, unlike hierarchical statistical features.

The comparison of fault severity for the broken fault is shown in Fig. 11b. The best performance was achieved by the composite hierarchical multi-scale approach for extracting non-linear information entropy features. This approach exhibits statistically significant differences in comparison to the composite multi-scale approach ($p=0.02$) and the hierarchical multi-scale approach used for extracting statistical features ($p=0.00$). In addition, the hierarchical multi-scale non-linear information entropy features show a statistically significant improvement over the hierarchical method for extracting statistical features ($p=0.02$). This comparison shows the RF model’s performance on the held-out test set.

Results of the comparison for the feature extraction methods applied to root crack fault severity are shown in Fig. 11c concerning the classification accuracy of the RF model. In this case, both hierarchical multi-scale and composite hierarchical multi-scale extraction methods achieved the best performance in extracting non-linear information entropy features for RF, evaluated on the held-out test set. Both methods exhibit statistically significant differences with respect to the composite multi-scale extraction method ($p=0.005$ and $p=0.000$) and the hierarchical method used to extract statistical features ($p=0.002$ and $p=0.000$).

The results comparing the scuffing fault severity are shown in Fig. 11d. Although the composite hierarchical multi-scale non-linear information entropy extraction method achieved the best performance, there were no statistically significant differences between the composite multi-scale and hierarchical multi-scale non-linear information entropy extraction methods. However, statistically significant differences are observed in the performance of CHMSE non-linear entropy features compared with the statistical features extracted using the hierarchical approach ($p=0.001$).

The composite hierarchical multi-scale method applied to non-linear information entropy feature extraction achieved the highest classification accuracy in the RF model across most fault severity types during evaluation on the held-out test set. In addition, their performance surpasses that of the hierarchical multi-scale statistical feature extraction method. Although the composite hierarchical multi-scale method is slightly more accurate than the hierarchical multi-scale method, it has the limitation that, for each signal in the hierarchical decomposition, the composite approach must be applied. The execution of the composite approach combined with the hierarchical approach substantially increases computational cost, especially when dealing with large signal windows. Given the absence of statistically significant differences between the hierarchical multi-scale and hierarchical composite multi-scale methods, the hierarchical multi-scale feature extraction method is more convenient for handling large signal windows.

An experiment was conducted to extract the selected non-linear information entropy feature set from AE signals, without employing any multi-resolution approach, and using the same signal window length as in all multi-scale experiments. The results are shown in Table 8 corresponding to the evaluation on the held-out test set of the RF model. The accuracy attained without applying any multi-scale approaches is in the column labeled *Non-linear Entropy*. The accuracy achieved with the hierarchical and composite hierarchical multi-scale approaches is consistently higher than that without any multi-scale approach for all fault types. The consistent 1–4% improvement of multi-scale methods over calculation without multi-resolution approaches demonstrates that fault severity information is distributed across temporal scales, which CHMSE effectively captures.

**Table 8:** Comparison of accuracy attained with the RF model. The column labeled as non-linear entropy list the results attained without applying any multi-scale approach.

**Fault Type**	**CMSE**	**Hierarchical MSE**	**Composite Hierarchical MSE**	**Non-linear Entropy**
Pitting	97.18	98.02	97.87	97.08
Broken	98.42	99.01	99.70	97.87
Crack	96.93	99.26	99.50	95.84
Scuffing	97.03	97.33	98.17	96.68

#### Comprehensive multi-metric performance analysis

While the previous analysis focused on classification accuracy, a more comprehensive evaluation of all six performance metrics–accuracy, sensitivity, specificity, precision, Kappa coefficient, and false positive rate (FPR)–reveals the breadth of the proposed method’s advantages.

**Fig. 12:**
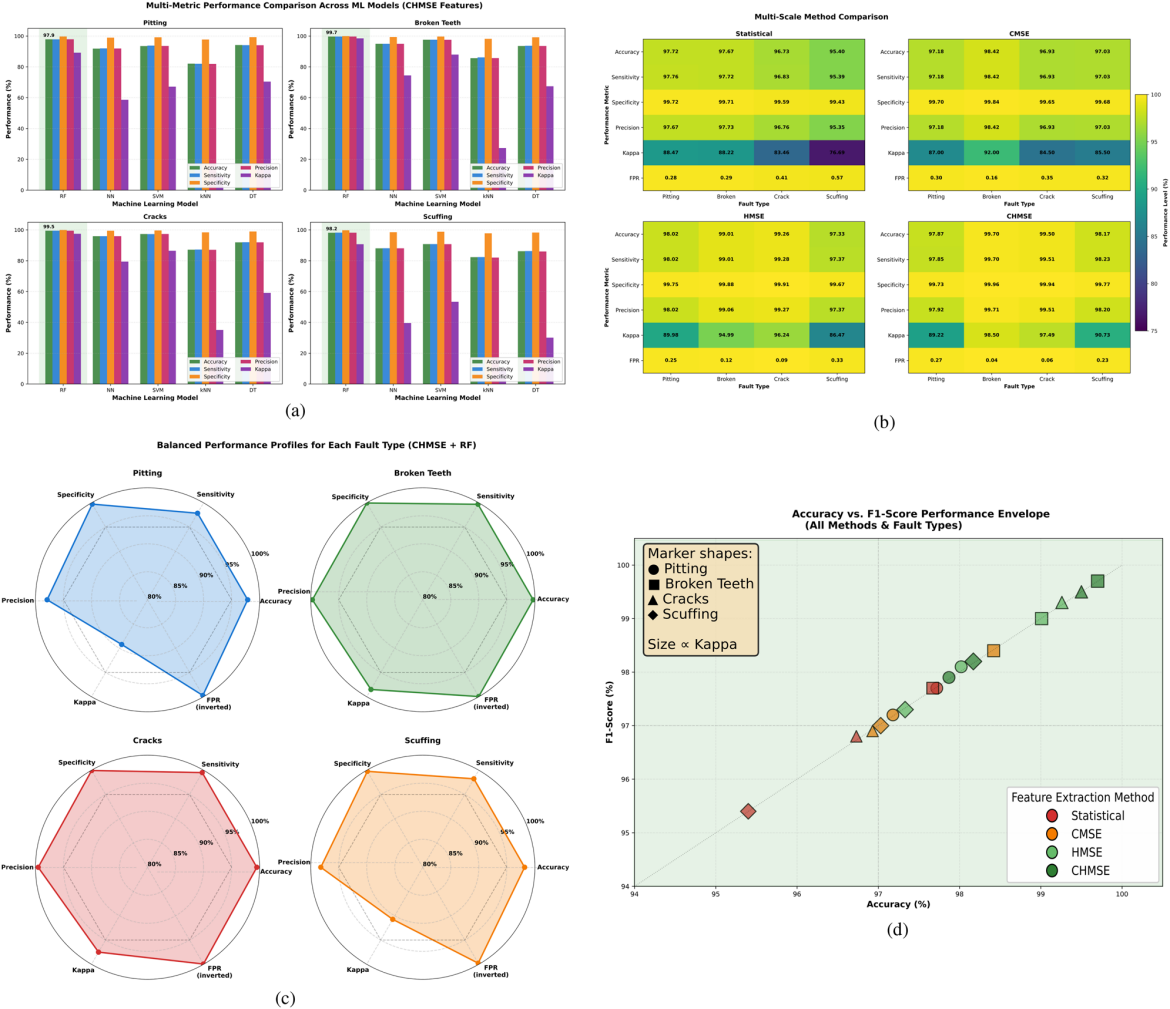
Comprehensive evaluation of all six performance metrics: accuracy, sensitivity, specificity, precision, Kappa coefficient, and false positive rate (FPR) obtained during the evaluation of each machine learning model on the held-out test set. The comparison shows the advantages of the hierarchical methods used in combination with non-linear entropy features for classification of fault severity. (**a**) Machine Learning Model Comparison, (**b**) Multi-Scale Method Comparison, (**c**) Balanced Performance Profiles, and (**d**) Performance Envelope Analysis.

- **Machine Learning Model Comparison**: Figure 12a shows the multi-metric performance comparison. Across all four fault types, Random Forests achieved superior performance not only in accuracy (97.87–99.70%) but also across all complementary metrics. Mean sensitivity ranged from 97.85% to 99.51%, indicating consistent fault-detection capability (high true-positive rates). Specificity values consistently exceeded 99.7% across all models and fault types, demonstrating strong resistance to false alarms. The Kappa coefficient for RF ranged from 89.22%–98.50%, indicating strong agreement beyond chance. In contrast, k-NN classifiers exhibited substantially lower performance across all metrics (Accuracy: 82–87%, Kappa: 9%−35%), confirming that distance-based classification is inadequate for this feature space. Neural Networks showed intermediate performance (Accuracy: 88–96%, Kappa: 39–79%).
- **Multi-Scale Method Comparison**: Figure 12b shows that the four feature extraction approaches (Hierarchical Statistical, CMSE, HMSE, CHMSE) across all metrics reveal consistent patterns. Hierarchical statistical features achieved acceptable accuracy (95.4–97.7%) but lower Kappa (76.7–88.5) and higher FPR (0.28–0.57%), suggesting greater misclassification of intermediate severity classes. CMSE entropy features improved accuracy by 0.5–1.0% and Kappa by 7–11 points. HMSE and CHMSE methods further improved performance, with CHMSE achieving the highest values across all metrics for broken teeth (Accuracy: 99.70%, Sensitivity: 99.70%, Specificity: 99.96%, Kappa: 98.50%, FPR: 0.04%) and cracks (Accuracy: 99.50%, Sensitivity: 99.51%, Specificity: 99.94%, Kappa: 97.49%, FPR: 0.06%). Significantly, performance improvements extended beyond accuracy; sensitivity and specificity ensure both reliable fault detection and minimal false alarms.
- **Balanced Performance Profiles**: Figure 12c shows the radar chart visualization of the best-performing configuration (CHMSE+RF) across the four fault types, demonstrating balanced, well-rounded performance across all metrics. Broken teeth and root crack severity classification achieved the most robust profiles, with all metrics exceeding 97.5% (except FPR, which remained below 0.10%). Pitting and scuffing maintained slightly lower Kappa coefficients (89–91%) compared to structural failures, yet still achieved excellent specificity (99.7–99.8%) and minimal FPR (0.23–0.27%). The similarity of the radar profiles across fault types indicates consistent diagnostic performance across different failure mechanisms.
- **Performance Envelope Analysis**: Figure 12d shows a scatter plot of the accuracy-F1-score relationship, revealing that CHMSE points consistently occupy the upper-right region (Accuracy> 97.5%, F1-Score > 97.5%) and have large Kappa values (point size), indicating simultaneous optimization across accuracy, precision-recall balance, and overall agreement. Statistical features scatter across lower regions with greater variability, while CMSE occupies intermediate positions. This envelope analysis confirms that CHMSE provides optimal multi-metric performance rather than optimizing a single metric at the expense of others.

**Fig. 13:**
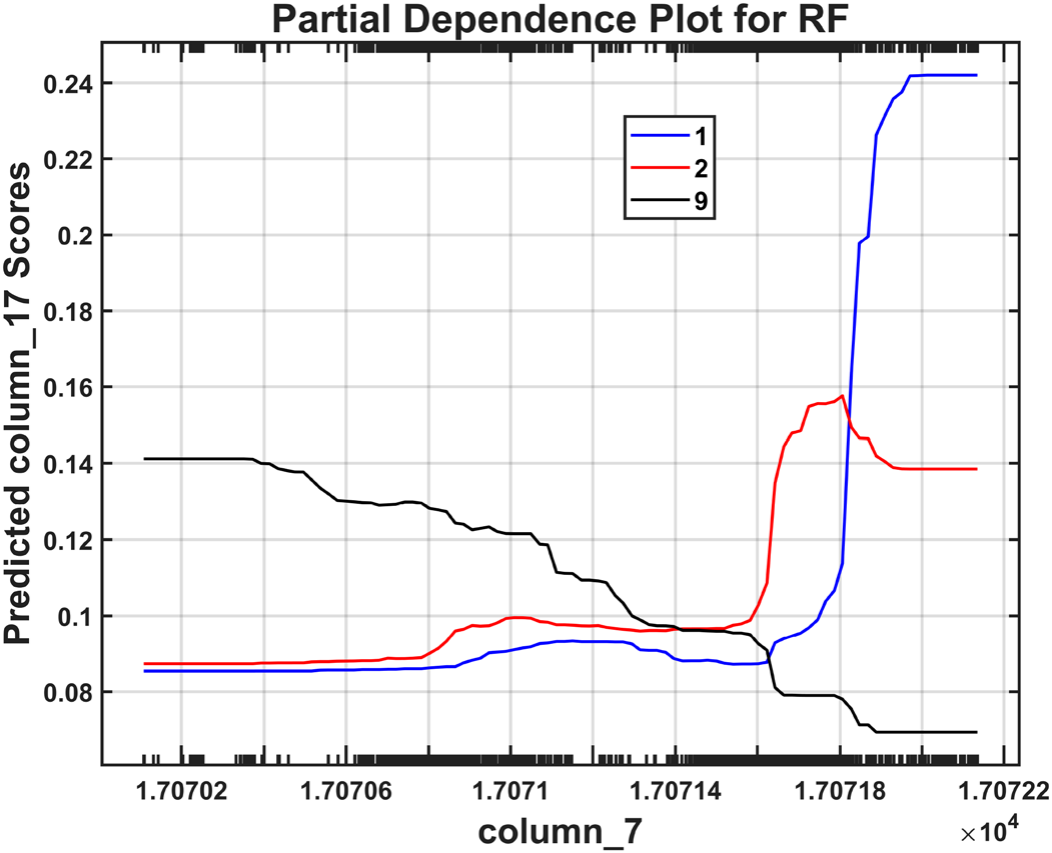
Shapley partial dependence for the RF model trained with the CHMSE features extracted from the pitting dataset. Dependence of classes 1, 2, and 9 on the feature 7 (threshold entropy). The healthy class (blue) shows a strong positive monotonic evolution where the increased threshold entropy values are related to higher prediction probabilities. Class 9 (black) shows an inverse relationship where higher threshold entropy values negatively contribute with this class prediction.

## Interpretability

Pitting fault severity is challenging to classify, and the best results from the RF model trained with the CHMSE features were an accuracy of 97.87%. Therefore, the interpretability of these results is discussed in the following paragraphs.

**Fig. 14:**
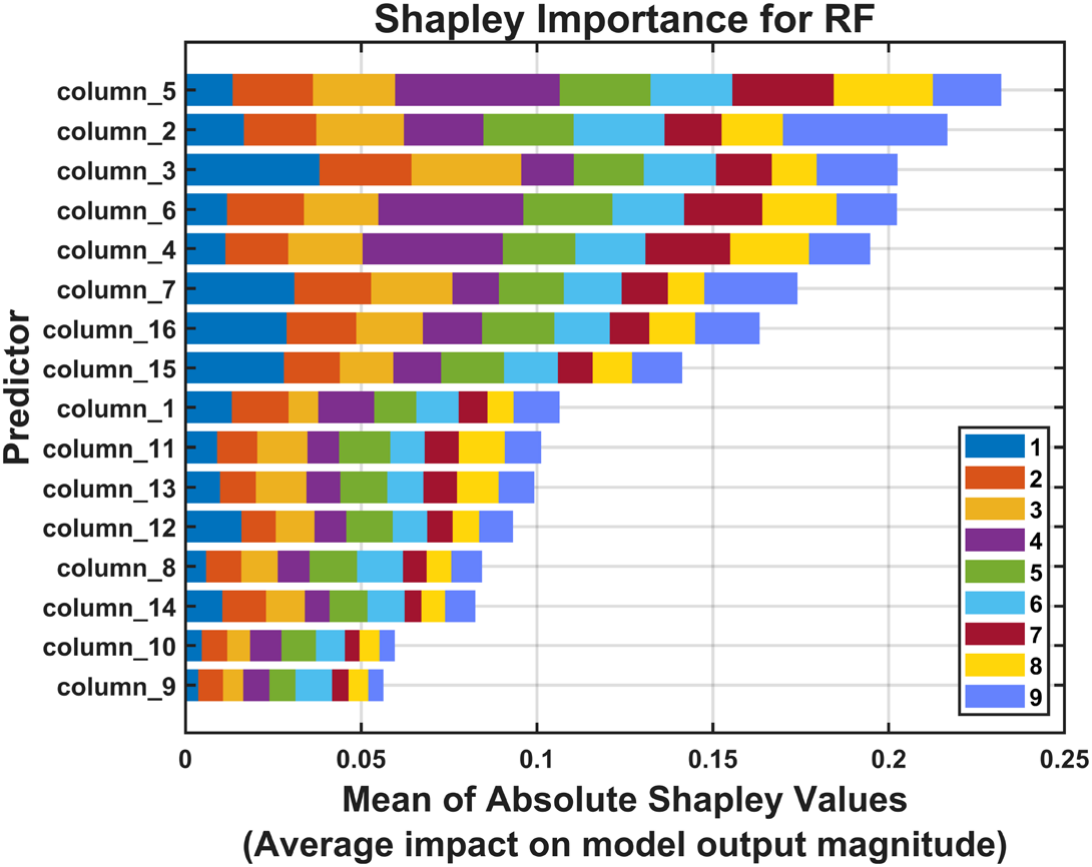
Shapley importance for the RF model trained with the CHMSE features extracted from the pitting dataset. The visualization identifies the CHMSE entropy features that exert the strongest influence on the random forest model classification decision. Feature 5 (log energy entropy, Table 1) emerges as the most important discriminator for pitting severity classification. Feature 2 (Rényi entropy) ranks as the second most influential feature.

Figures 13,14 reveal which entropy features drive classification decisions across nine severity levels (P1-P9) and how these features interact with fault progression.

Figure 13 presents the partial dependence plots for feature 7 (threshold entropy) across three representative severity classes (P1, P2, and P9), showing how this feature’s values affect the Random Forest model’s predictions while averaging over all other features. These plots reveal distinct and contrasting functional relationships that enable robust discrimination among severity levels: the healthy state (class 1, blue curve starting at the left lower end and growing to maximum at the right top) exhibits a strong positive monotonic relationship where increasing threshold entropy values correspond to higher prediction probabilities; conversely, the advanced fault state (class 9, black curve decreasing from left to right) demonstrates an inverse relationship where higher threshold entropy values negatively contribute to this class’s predictions; while the incipient fault state (class 2, red curve) maintains an intermediate behavior positioned between these extremes. This opposing behavior between healthy and severely damaged conditions suggests that threshold entropy–derived from wavelet packet decomposition of acoustic emission signals–captures fundamental changes in signal complexity as pitting damage progresses, with healthy gears generating acoustic emissions of higher entropy and severely damaged gears producing more deterministic, lower-entropy patterns dominated by periodic impacts from damaged tooth surfaces. The clear separation and opposing slopes of these partial dependence curves provide interpretable, physics-consistent evidence that CHMSE-extracted entropy features enable the Random Forest classifier to establish well-defined decision boundaries across the fault severity spectrum.

Figure 14 presents the global feature importance ranking based on mean absolute SHAP values across all test predictions for the pitting fault severity dataset. This visualization identifies which CHMSE features exert the strongest influence on Random Forest classification decisions. Feature 5 (log energy entropy, Table 1) emerges as the most important discriminator for pitting severity classification. Feature 2 (Rényi entropy) ranks second among the most influential features. In addition, features 3 and 6 (Tsallis and norm entropy) are also of substantial importance, as are features 4 (Shannon entropy) and 7 (Threshold entropy). The prominence of threshold entropy (feature 7) suggests that amplitude-based entropy thresholds effectively capture the transient acoustic emission events generated when pitted gear teeth engage during meshing cycles. Threshold entropy, which measures information content relative to signal amplitude thresholds ($p = 3.5 \times std(x)$), is particularly sensitive to the intermittent bursts characteristic of surface pitting defects. Tsallis entropy (feature 3), a generalized entropy measure with parameter $q_T=1.5E-5$, exhibits greater sensitivity to rare events than Shannon entropy. This property makes it well-suited for detecting incipient pitting where fault signatures manifest as infrequent, high-amplitude transients embedded in background operational noise. The importance of spectral entropy derivatives features (features 15–16) indicates that frequency-domain complexity patterns–specifically the statistical distribution characteristics (skewness and kurtosis) of instantaneous spectral entropy–carry critical information about pitting progression. Progressive surface degradation alters both the amplitude distribution (skewness) and the presence of outlier events (kurtosis) in the spectral entropy time series^101^.

**Fig. 15:**
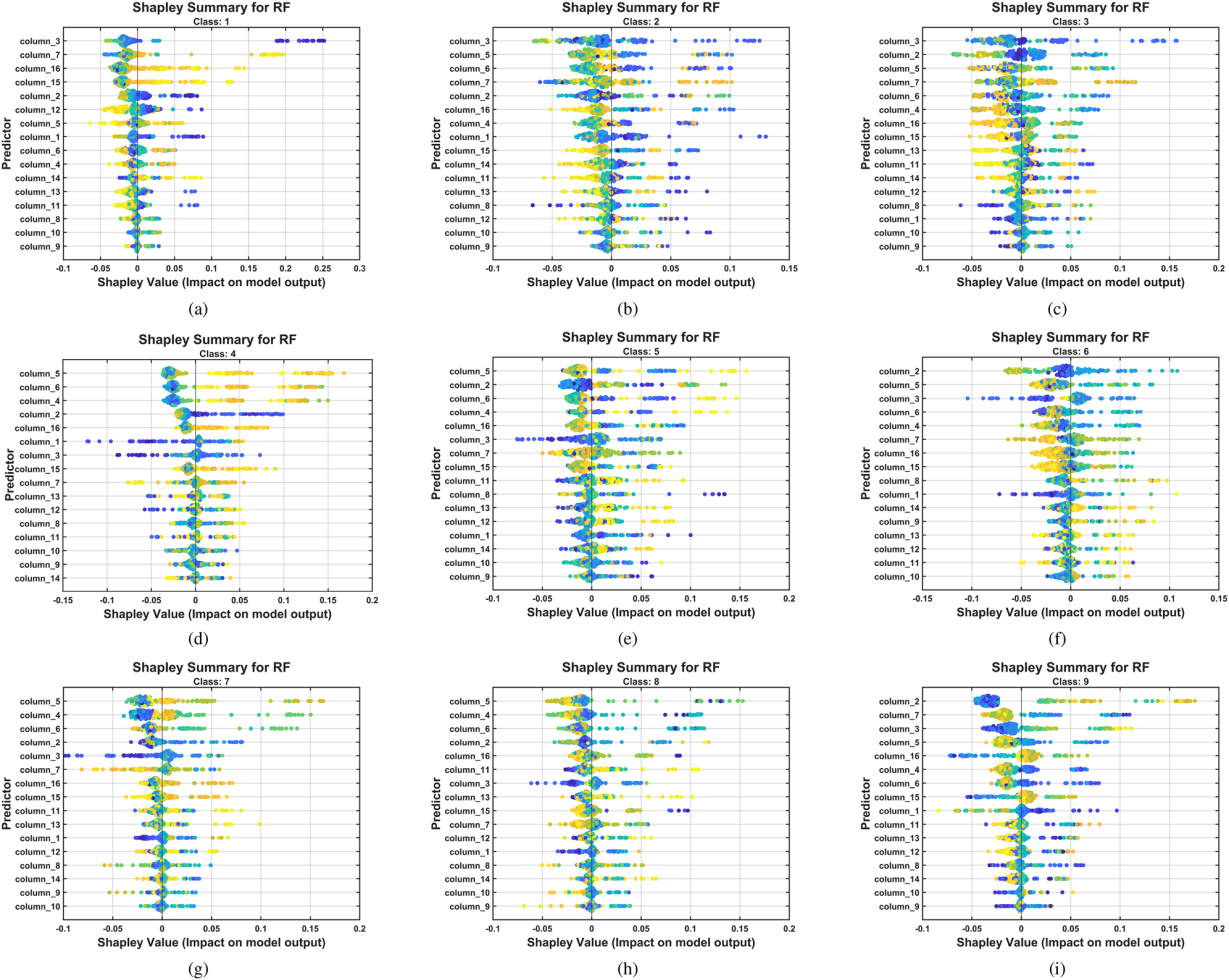
Shapley summary for the RF model trained with the CHMSE features extracted from the pitting fault severity. The beeswarm plots show features in the vertical axis ranked by their importance for predicting the specific class. The SHAP values are shown on the horizontal axis, indicating each feature’s contribution to the particular class prediction. (**a**) Class 1, (**b**) class 2, (**c**) class 3, (**d**) class 4, (**e**) class 5, (**f**) class 6, (**g**) class 7, (**h**) class 8, (**i**) class 9.

Figure 15 presents nine SHAP summary plots (panels a-i), one for each pitting severity level (Classes P1-P9). These beeswarm plots display features on the vertical axis (ranked by their importance for predicting that specific class), SHAP values on the horizontal axis (indicating each feature’s contribution to the prediction), and feature value magnitude represented by color (red=high, blue=low). Healthy (P1) and severe (P9) conditions show more concentrated SHAP value distributions along the horizontal axis (indicating consistent feature contributions across samples). In contrast, intermediate classes show wider horizontal spreads (indicating greater sample-to-sample variability in feature contributions).

**Fig. 16:**
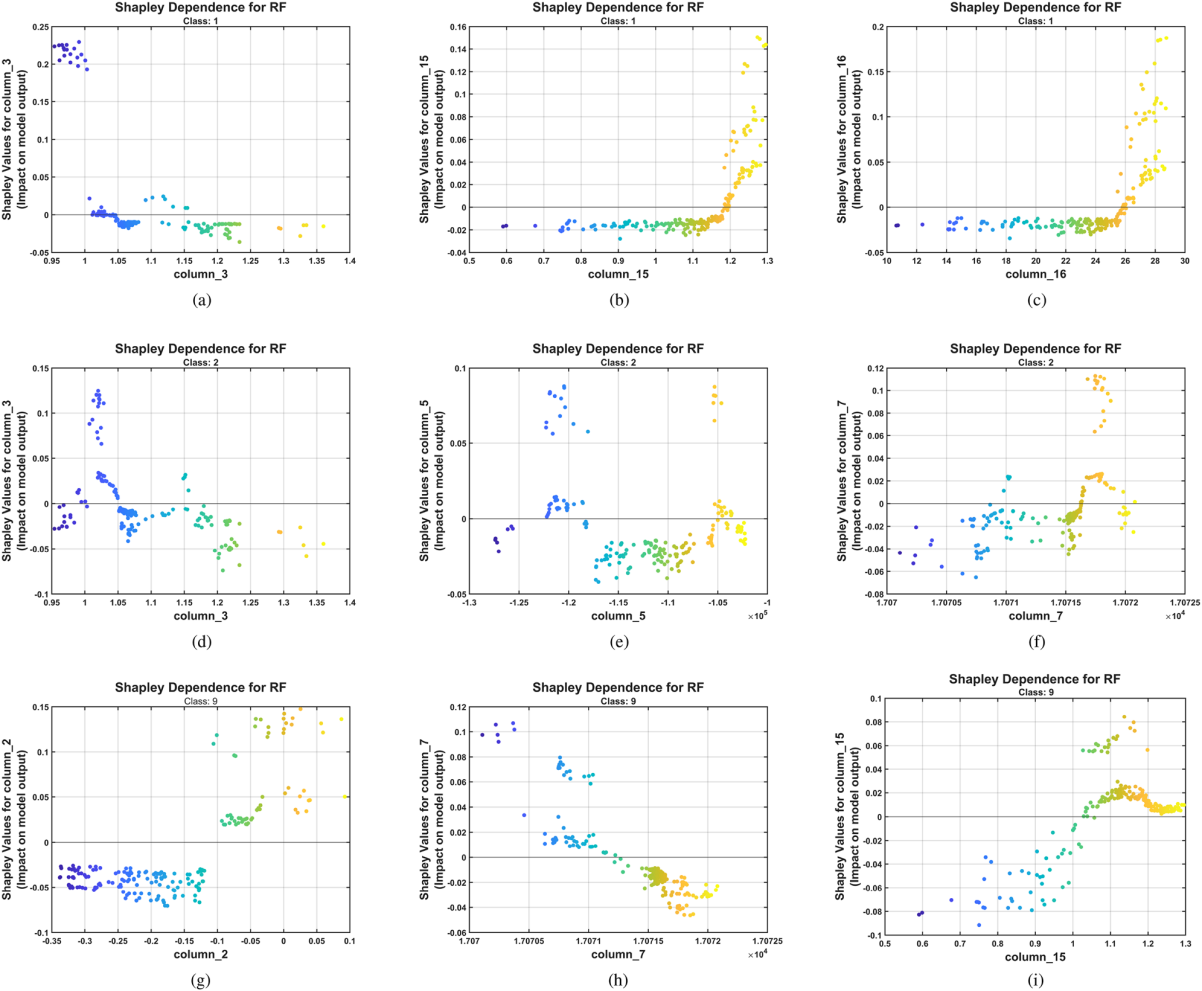
Shapley dependence for the RF model trained with the CHMSE features extracted from the pitting fault severity. The plots show how specific feature values relate to the SHAP contribution for three representative severity classes (1, 2 and 9). (**a**) Class 1 dependence on feature 3 (Tsallis entropy), (**b**) class 1 dependence on feature 15 (skewness), (**c**) class 1 dependence on feature 16 (kurtosis), (**d**) class 2 dependence on feature 3 (Tsallis), (**e**) class 2 dependence on feature 5 (log energy entropy), (**f**) class 2 dependence on feature 7 (threshold entropy), (**g**) class 9 dependence on feature 2 (Rényi entropy), (**h**) class 9 dependence on feature 7 (threshold entropy), (**i**) class 9 dependence on feature 15 (skewness).

Figure 16 presents nine dependence plots showing how specific feature values relate to SHAP contributions for three representative severity classes (1, 2, and 9): the class 1 (Healthy) is shown in panels a, b, c, class 2 in panels (d, e, f), and class 9 in panels (g, h, i). The vertical dispersion patterns observed in these plots indicate feature interactions, in which the impact of one feature on the prediction depends on the values of other features. This phenomenon has been extensively documented in explainable AI literature for machinery fault diagnosis applications^102^. Notably, feature 7 (threshold entropy) and feature 3 (Tsallis entropy) exhibit distinct clustering behaviors across different severity levels, with tighter, more concentrated clusters in the healthy state (P1) transitioning to more dispersed distributions in advanced fault states (P9), suggesting that increased scatter reflects the greater variability in acoustic emission characteristics as pitting damage progresses. Importantly, the clusters for the healthy state (P1) maintain substantial absolute SHAP values, indicating that these features contribute significantly to correct classification across all severity levels, not merely for damaged conditions. This visualization provides transparent evidence that the proposed CHMSE-extracted features enable the Random Forest classifier to learn physically meaningful decision boundaries consistent with the progressive nature of pitting damage mechanisms in gearbox systems.

**Fig. 17:**
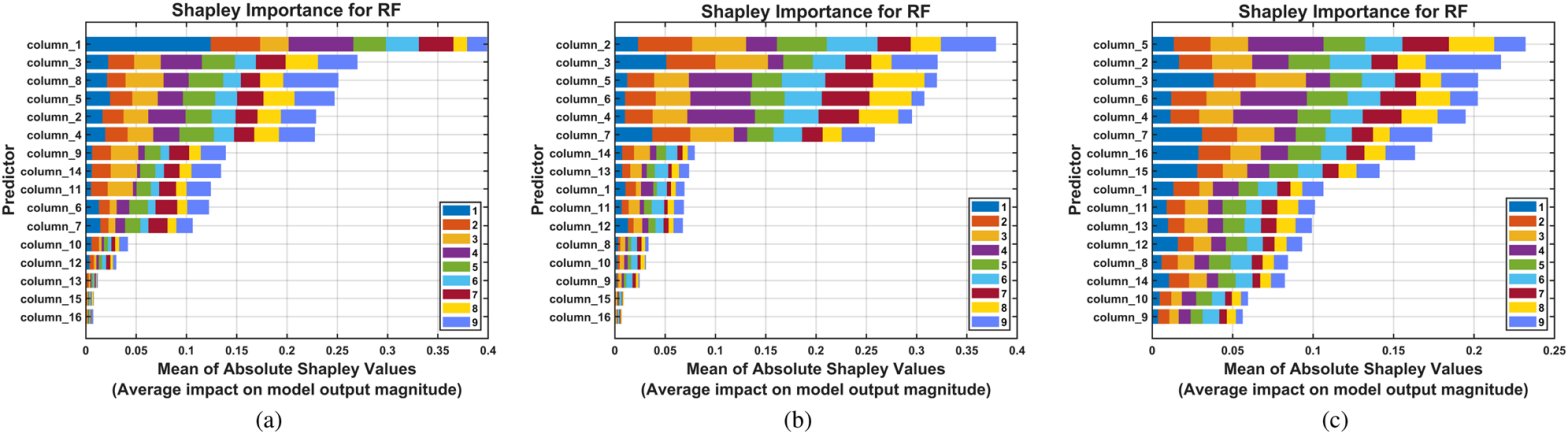
SHAP feature importance rankings across multi-scale methods. (**a**) CMSE reveals log energy entropy as dominant discriminator; (**b**) HMSE demonstrates importance of generalized entropy measures (Rényi, Tsallis) and wavelet packet features (threshold entropy, norm entropy); (**c**) CHMSE shows balanced multi-feature utilization with similar top-ranked features as HMSE, indicating that hierarchical decomposition unlocks wavelet packet entropy discriminative power.

## Discussion

### Principal findings and their interpretation

This study demonstrates that composite hierarchical multi-scale entropy combined with non-linear information entropy features achieves superior classification performance for acoustic emission-based fault severity diagnosis in spur gearboxes. Across all four fault types examined–pitting, broken teeth, root cracks, and scuffing–the CHMSE approach consistently outperformed conventional multi-scale methods and statistical feature extraction when paired with Random Forest classifiers.

Feature importance analysis across the three multi-scale approaches (Fig. 17) reveals consistent patterns that illuminate the mechanisms underlying CHMSE’s superior performance. Specifically, a core set of generalized information entropy features demonstrated critical importance for Random Forest classification when combined with both CHMSE and HMSE decomposition methods (see Table 1): Rényi entropy (feature 2) and Tsallis entropy (feature 3), along with Shannon information entropy (feature 4) and log energy entropy (feature 5). These second-order entropy measures provide balanced sensitivity to both typical and rare events through their generalized-entropy formulation, effectively capturing the non-Gaussian distributional characteristics inherent in acoustic emission signals during progressive fault development. Wavelet packet-based entropy features–including norm entropy (feature 6) and threshold entropy (feature 7)–exhibited substantial discriminative power for CHMSE and HMSE approaches, yet demonstrated markedly reduced importance when extracted using CMSE decomposition alone. This differential behavior suggests that hierarchical decomposition’s preservation of high-frequency transient components via complementary operators ($Q_0$ and $Q_1$) is essential for wavelet packet methods to capture fault-related information across multiple temporal scales effectively. Overall, the consistency of core entropy feature rankings across hierarchical methods (HMSE and CHMSE) demonstrates that multi-scale temporal decomposition fundamentally enhances the discriminative capacity of information-theoretic measures, regardless of whether composite averaging is applied at each scale.

Comparative analysis of SHAP value distributions across multi-scale methods reveals distinct feature importance patterns (Fig. 17). HMSE demonstrated more concentrated SHAP value distributions with sharper separation between wavelet packet-based entropy features (features 5–8) and spectral entropy features (features 9–16). By contrast, CHMSE exhibited a more gradual importance gradient, ranging from log energy entropy (feature 5) as most important to mean spectral entropy (feature 9) as least important. This gradual distribution pattern reflects balanced multi-feature utilization, enabling CHMSE to achieve superior classification performance through composite hierarchical decomposition. CMSE displayed intermediate separability, confirming that composite averaging in CHMSE regularizes feature importance while maintaining discriminative capability.

The superior performance of CHMSE can be attributed to its dual advantages in addressing fundamental limitations of standard multi-scale approaches. First, the hierarchical decomposition preserves both low-frequency and high-frequency components of the acoustic emission signals through the complementary operators $Q_0$ and $Q_1$^59^. In contrast, conventional coarse-graining approaches in standard MSE effectively perform low-pass filtering, discarding high-frequency fault signatures^82^. This low-pass filtering is particularly critical for acoustic emission signals, which contain transient high-frequency components generated by crack formation, material loss, and surface interactions^20,46^. Second, the composite approach reduces variance in entropy estimation by averaging features across multiple coarse-grained time series at each scale^58^, thereby improving stability and generalization performance, especially for intermediate-severity classes that exhibit the most classification errors.

The composite hierarchical multi-scale method achieved remarkable accuracy (99.50% for root cracks, 99.01% for broken teeth), representing substantial improvements over the composite multi-scale approach alone. Statistical analysis confirmed these differences were significant for broken tooth ($p = 0.02$) and root crack ($p < 0.01$) classifications. Interestingly, for scuffing faults, the performance differences among the three multi-scale approaches were not statistically significant, suggesting that the discriminative information for scuffing severity may be more uniformly distributed across frequency scales, making all multi-scale approaches comparably effective.

### Comparison with existing literature

Utilization of deep learning approaches for fault severity classification is scarce. Even the classification of advanced fault stages using deep learning in combination with acoustic emission signals is not very common. The research reported in^27^ describes a multi-input convolutional neural network used to fuse vibration and acoustic emission signals for the classification of seven fault types in a milling machine. In^28^, a new MEMS acoustic emission sensor is developed and tested for the classification of five fault conditions in gears using a ResNet-18 convolutional neural network. In both cases, to deal with the high frequency of the acoustic emission signal, the approach preprocesses the AE signal to obtain a time-frequency representation, which is then fed to the convolutional neural network for fault classification. Recent research combining 1-D Convolutional neural networks with bi-directional Long Short-Term Memory (LSTM) neural networks has been reported in^29^ for the classification of the content of brittle minerals in rocks. Although interesting, the approach has not been applied to rotating machinery. A recent study reported in^30^ applies a 1D-CNN architecture to gearbox fault detection using low-frequency vibration signals and signal windows of only 120 samples. The 120-sample window approach successfully applied to vibration signals in^30^ is unsuitable for Acoustic Emission (AE) monitoring. At a 1 MHz sampling rate, such a window covers only 120 $\mu$s, which is insufficient to capture the complete transient profile of an AE burst–typically lasting between 500 $\mu$s and 2 ms. Increasing the window size to capture these events would lead to high-dimensional input vectors, imposing significant computational overhead on 1D-CNN architectures due to the increased number of operations required for long-sequence feature extraction.

**Fig. 18:**
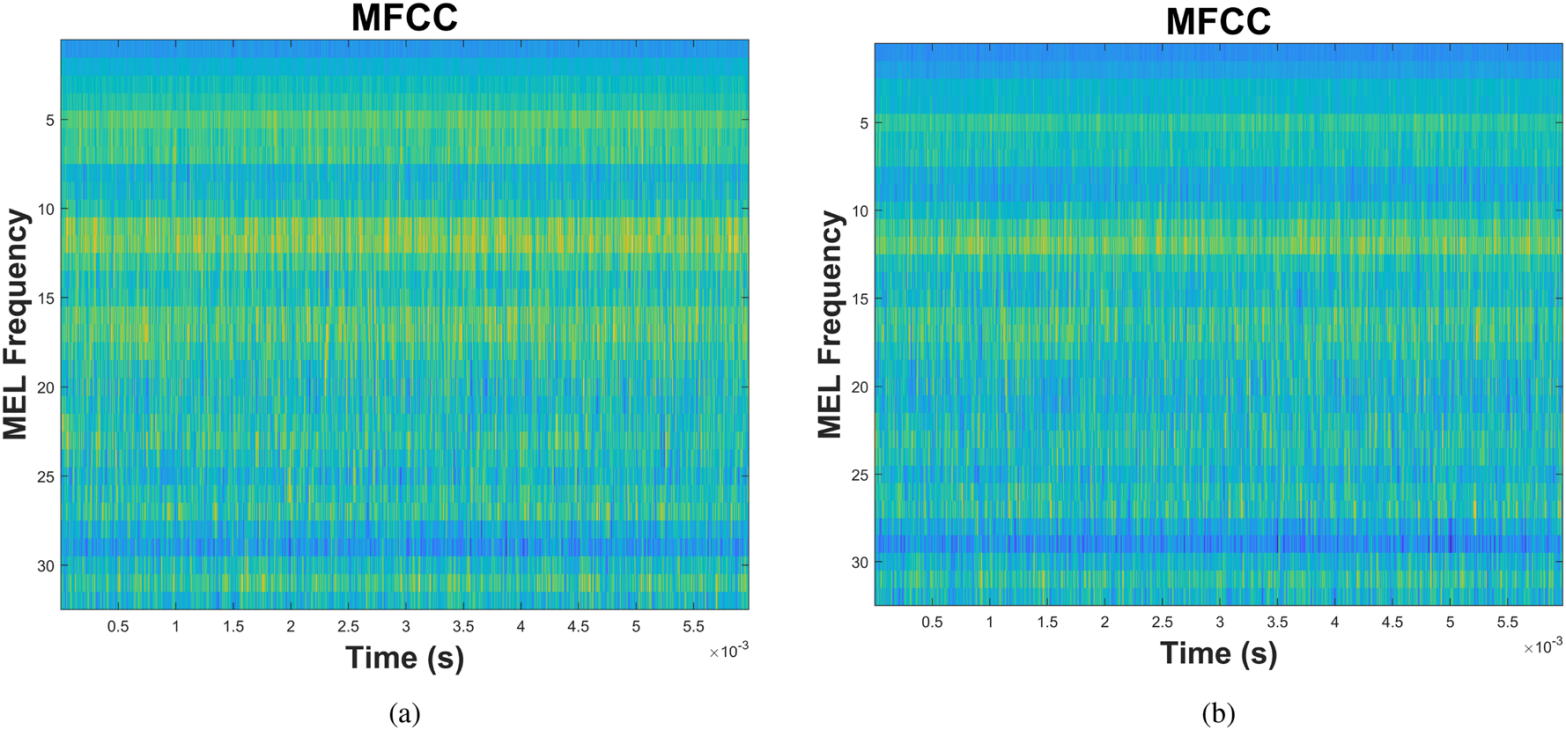
Mel frequency spectrum coefficients for an acoustic emission signal acquired from the spur gearbox. (**a**) MFCC for a signal from the healthy class P1, (**b**) MFCC for a signal from the P9 pitting fault severity dataset.

In this research, we have performed a test using a deep learning approach to compare it with the proposed methodology. Specifically, we have used a convolutional neural network fed with a time-frequency representation of the acoustic emission signal. The time-frequency representation used is the Mel Frequency Cepstrum Coefficients (MFCC), which has been previously reported in combination with sound or vibration signals^103^. The AE MFCC was obtained using an overlapped window of 10 ms with a stride of 0.5 ms and 50 Mel frequency bands for the Mel filter-bank. The *delta* array and *delta-delta* array were also calculated and concatenated. The feature array has a size of $96\times 5981$. The MFCC array of size $32 \times 5981$ is shown in Fig. 18 for an AE signal extracted from the healthy condition and for the advanced pitting fault severity P9. Subtle differences in the content of both arrays are visible, mainly related to the location and concentration of the events. Using an array of size $96 \times 5981$ in combination with any CNN imposes significant constraints on computational cost. It was necessary to use a highly efficient approach based on the feature array’s subdivision. The CNN architecture and details concerning the deep learning approach are reported in^104^.

**Fig. 19:**
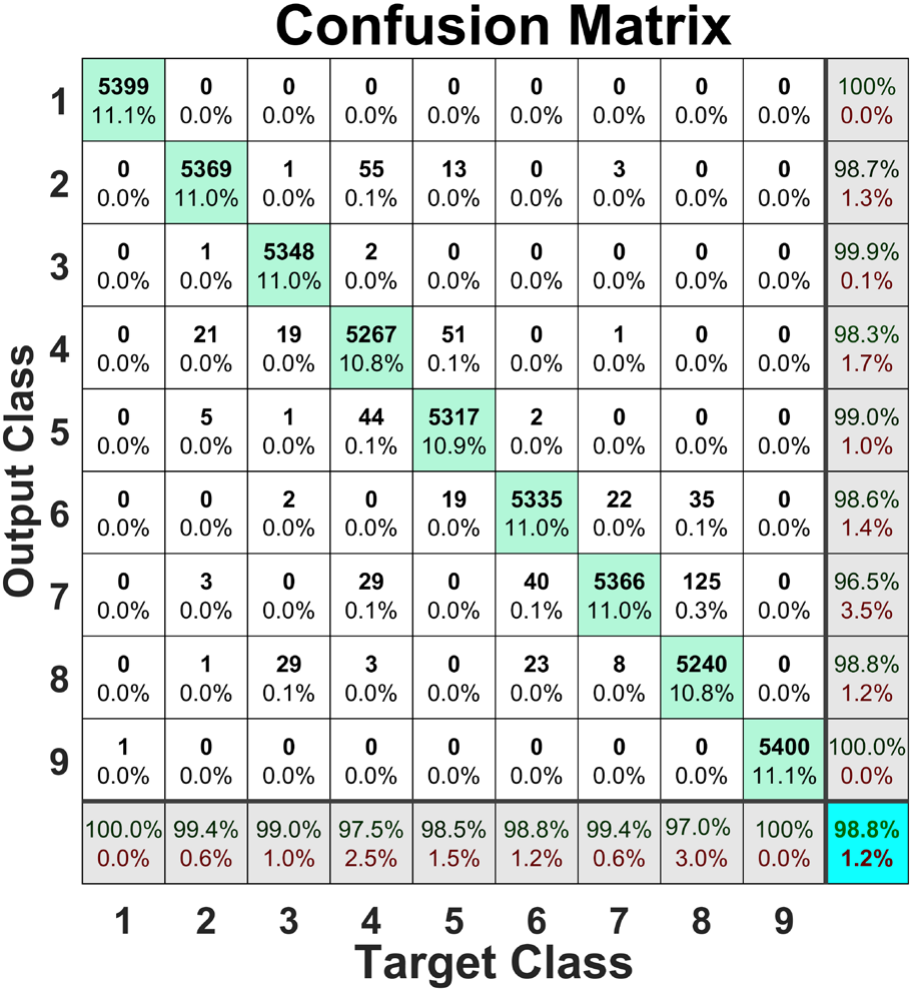
Confusion matrix obtained for the classification of pitting fault severity using MFCC features as the input of a Convolutional Neural Network.

The approach was tested with the pitting fault severity dataset. The confusion matrix obtained is shown in Fig. 19. The overall performance metrics are listed in Table 9, obtaining an average classification accuracy of 98.8%. This represents improvements of 1.62% for the CMSE features, 0.78% for the HMSE, and 0.93% for the CHMSE non-linear entropy features using the RF model. The performance metrics concerning each of the pitting fault severity classes are presented in the Table 10 and demonstrate an excellent diagnostic capability of the MFCC-CNN framework in classifying spur gearbox pitting severity. The model achieved near-perfect classification across several states, particularly for classes 1 and 9, where F1-scores reached 99.99%, indicating strong discriminative power at the boundaries of the severity spectrum. Sensitivity remained high across all categories, with class 7 representing the lower bound at 96.46%, suggesting that even the most challenging fault signatures were successfully captured. Furthermore, the low False Positive Rates (FPR), all remaining below 0.38%, and Error Rates consistently under 0.5%, confirm the model’s reliability in preventing misclassification between adjacent severity levels. These results suggest that the Mel-frequency cepstral coefficients effectively compressed 1 MHz acoustic emission signals into representative feature maps, enabling the CNN to distinguish subtle morphological changes in fault signatures with high precision and specificity.

**Table 9:** Overall Performance Metrics for MFCC-CNN approach for pitting fault severity classification using AE signals (in percent).

**Accuracy**	**Error Rate**	**Sensitivity**	**Specificity**	**Precision**	**F1-Score**
98.82	1.18	98.86	99.86	98.85	98.85

**Table 10:** Per-Class Performance Metrics in percents concerning the results of the MFCC-CNN approach.

**Class**	**Sensitivity (Recall)**	**Specificity**	**Precision (PPV)**	**F1-Score**	**FPR**	**Error Rate**
1	100.00	99.99	99.98	99.99	0.00	0.00
2	98.68	99.93	99.43	99.05	0.07	0.21
3	99.94	99.88	99.04	99.49	0.12	0.11
4	98.27	99.69	97.54	97.90	0.31	0.47
5	99.03	99.81	98.46	98.75	0.19	0.28
6	98.56	99.85	98.78	98.67	0.15	0.30
7	96.46	99.92	99.39	97.91	0.08	0.47
8	98.79	99.63	97.02	97.90	0.37	0.46
9	99.99	100.00	100.00	99.99	0.00	0.00

Our results align with and extend previous findings on multi-scale entropy approaches for rotating machinery diagnostics. Chen et al.^56^ reported 100% accuracy for gearbox fault classification using improved multi-scale amplitude-aware permutation entropy combined with Intrinsic Time Decomposition, albeit for a more straightforward five-condition classification task using vibration signals. Our study achieves comparable performance (99.70% for broken teeth). However, it addresses the more challenging problem of severity classification across nine levels using acoustic emission signals, which are inherently more complex and susceptible to attenuation.

Similarly, Wei et al.^59^ demonstrated the effectiveness of refined composite hierarchical fuzzy entropy for planetary gearbox fault diagnosis. Our work extends this approach by systematically comparing multiple entropy-based features and showing that information entropy features–which are computationally less intensive than fuzzy entropy–can achieve comparable or superior classification performance. This computational advantage is particularly relevant for real-time condition monitoring applications, where processing efficiency is critical.

Unlike most reported studies that focus on vibration signals^56,57^, our research explicitly addresses acoustic emission signals for multi-scale entropy analysis. While AE signals offer earlier fault detection capabilities^6,7^, they present unique challenges, including high sampling rates (1 MS/s in our study), rapid attenuation, and strong non-linearity^22^. The success of our approach in handling these challenges suggests that multi-scale non-linear information entropy methods are robust feature extraction techniques for high-frequency, non-stationary signals.

### Feature set selection and computational efficiency

**Fig. 20:**
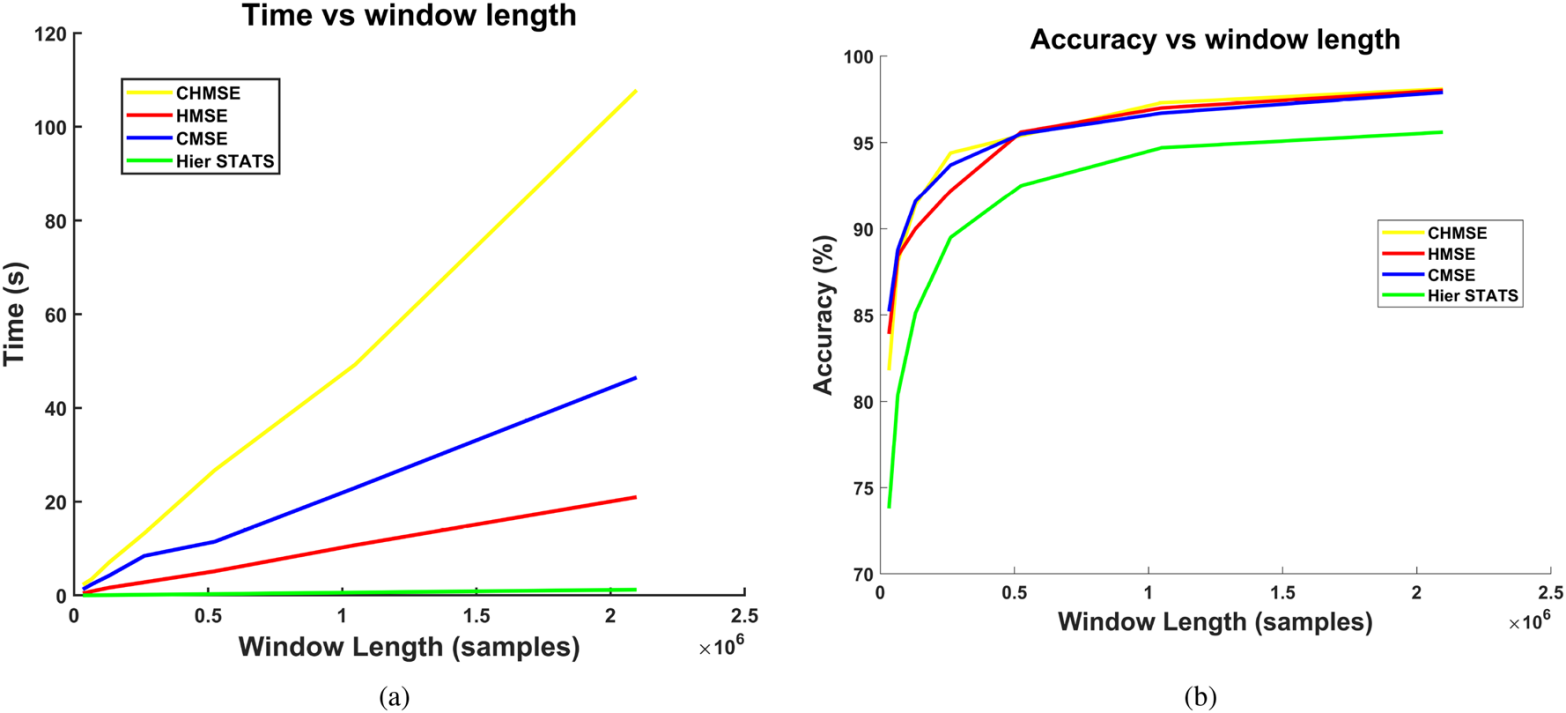
Estimated computational time and accuracy as a function of the window length. (**a**) Computational time as a function of the window length, (**b**) Accuracy as a function of the window length.

In this work, the methodology for feature extraction and classification was implemented on MATLAB software running on a laptop computer with a processor Intel i7-6700HQ @ 2.59 GHz with 12 GB of RAM. The time required to extract the non-linear information entropy features from a signal with a window length of 2$^{21}$ samples was 72.40 s using the CMSE method, 25.11 s using the HMSE method, and 130.17 s using the CHMSE. Interestingly, entropy calculations that rely on distance metrics are considerably higher. For instance, calculating the sample entropy using the CMSE approach with a window of length $2^{18}=262144$ samples takes 433.91 s. A comparison concerning the computational time in the specified hardware as a function of the window length is shown in Fig. 20a. The computational cost increases linearly with the window length, and the RF model trained with the HMSE non-linear information entropy feature set showed the highest efficiency. In contrast, the RF model trained with CHMSE had the lowest computational efficiency. Although extracting hierarchical statistical features is computationally efficient, their accuracy is lower than that of non-linear entropy features. In Fig. 20b, the variation of the accuracy with the window length is shown. The accuracy grows slowly for window lengths greater than 500,000 samples. However, for shorter window lengths, accuracy decreases significantly across all feature types and multi-scale extraction methods.

A critical contribution of this work is the demonstration that computationally efficient information entropy features can match or exceed the performance of more complex non-linear features that rely on phase space reconstruction and distance calculations. The selected feature set (Table 1) combines permutation entropy, Rényi entropy, Tsallis entropy, wavelet packet entropy variants, and spectral entropy derivatives.

This computational advantage enabled us to analyze extended signal windows (2$^{21}$ = 2,097,152 samples) without prohibitive processing times. The comparison with hierarchical multi-scale statistical features (Table 3) reveals that non-linear information entropy features consistently provided 1–4% accuracy improvements across all fault types. This improvement in accuracy suggests that capturing the complexity and irregularity of AE signals through entropy measures better discriminates fault severity levels than amplitude-based statistical descriptors alone.

However, the performance gap between entropy and statistical features was relatively modest, particularly for pitting faults (98.02% vs. 97.72%). This modest performance gap indicates that for specific fault mechanisms, the temporal and spectral patterns captured by statistical features substantially overlap with the complexity patterns captured by entropy measures^105^. Further investigation into the complementarity of these feature types could yield insights for optimal feature fusion strategies.

The computational requirements of the proposed methods must be evaluated in the context of industrial condition monitoring practices. Gearbox fault progression is a gradual phenomenon, typically evolving over days to weeks rather than minutes^106^. Consequently, practical monitoring systems employ periodic data acquisition schedules–hourly, per-shift, or daily–rather than continuous streaming analysis. The relevant computational constraint is therefore whether feature extraction can be completed within the interval between successive measurements, not whether processing speed matches the 1 MHz signal acquisition rate^107^.

Under this practical definition of real-time capability, all three methods are viable for industrial deployment. HMSE, with a processing time of 25.11 seconds per signal on standard laptop hardware, enables analysis of 143 signals per hour–sufficient for monitoring multiple gearboxes or acquiring redundant measurements within each cycle. Even CHMSE, which requires 130.17 seconds, allows processing 28 signals per hour, compatible with typical hourly monitoring schedules, while providing enhanced classification accuracy.

For industrial implementation, we recommend a method selection strategy based on operational requirements. For continuous online monitoring where computational resources are constrained, or multiple assets require simultaneous analysis, HMSE provides the optimal balance of efficiency and accuracy–its performance is statistically equivalent to CHMSE for most fault types while requiring only 19% of the computational time. For critical diagnostic assessments or post-incident root cause analysis where maximum accuracy is paramount and processing delays are acceptable, CHMSE is preferred. A hybrid deployment strategy may also be effective: HMSE for routine screening across all monitored assets, with CHMSE analysis triggered selectively when anomalies are detected, thereby optimizing computational resources while maintaining diagnostic depth for flagged conditions.

The reported computational times reflect MATLAB implementation on a laptop computer (Intel i7-6700HQ @ 2.59 GHz, 12 GB RAM). Substantial performance improvements are achievable through optimized implementations: compiled C/C++ code typically achieves 5–10$\times$ speedups over interpreted MATLAB, and GPU acceleration can further reduce processing time for parallelizable entropy calculations. Such optimizations would enable CHMSE processing in approximately 13–26 seconds, expanding its applicability to more demanding monitoring scenarios, including near-real-time diagnostic feedback systems^108^.

### Parameters selection

A comprehensive list of parameters is presented in Table 11. The description and justification of these parameters are presented in the following paragraphs.

**Table 11:** Selected parameter configuration for multi-scale entropy analysis.

**Parameter**	**Symbol**	**Value**	**Justification**
Analysis window length	–	$2^{21}$ samples	FFT efficiency and multi-scale entropy reliability
Rényi entropy	$q_{R}$	0.5	Optimized via grid search (Figure 21a)
Tsallis entropy	$q_{T}$	$1.5\times 10^{-5}$	Optimized via grid search (Figure 21b)
Hierarchical layers	*k*	3	Balance between scale coverage and data length
CMSE scales	*s*	2	Computational efficiency vs. accuracy
Norm entropy	$p_N$	1.8	Optimized via grid search (Figure 21c)
Threshold entropy	$p_{Thres}$	3.5	Optimized via grid search (Figure 21d)
Sure entropy	$p_S$	3.5	Optimized via grid search (Figure 21e)
Permutation entropy	*m*	3	Computational efficiency and accuracy (Figure 21f)

#### Analysis window length

The window length was chosen based on the results regarding accuracy as a function of window length. These results are shown in Fig. 20, where the accuracy grows slowly to 95% for window lengths greater than 500,000 samples. The choice of a window length of $2^{21}$ samples provides several analytical advantages: the power-of-two length enables efficient Fast Fourier Transform (FFT) computation and wavelet packet decomposition without zero-padding, which is critical for the spectral entropy and wavelet packet entropy calculations employed in this study. In addition, the multi-scale entropy methods require sufficiently long time series to provide reliable estimates of information entropy across multiple scales. Concerning the experimental gearbox operating at speeds of 900, 1200, and 1500 RPM (15–25 Hz shaft rotation), a 2.097-second window captures 31–52 complete shaft rotations and 527–878 gear mesh cycles. This duration is sufficient to characterize both periodic fault signatures and their modulation patterns.

#### Multi-scale entropy parameters

The scale Factor Range was selected as *s*= 1 to 2 based on Signal Length Constraints. Specifically, after coarse-graining at scales, the effective time series length is *N*/*s*. With an analysis window of about 2.1 million samples, the scale *s*=2 yields a practical series of more than 1,048,576 samples, which remains sufficient for reliable entropy estimation. Using higher values of s increases the computational cost. Concerning the frequency Coverage, at a sampling rate of 1 MS/s, a scale of *s* = 2 corresponds to analyzing the frequency content down to approximately 250 kHz. This range adequately captures the acoustic emission content characteristic of gear-fault mechanisms. Finally, the scale range *s* = 1 to 2 aligns with established practices in multi-scale entropy analysis for machinery condition monitoring, where typical ranges of *s* = 1 to 15 have been effectively employed for vibration and acoustic emission signal analysis^109^.

#### Permutation entropy parameters

**Fig. 21:**
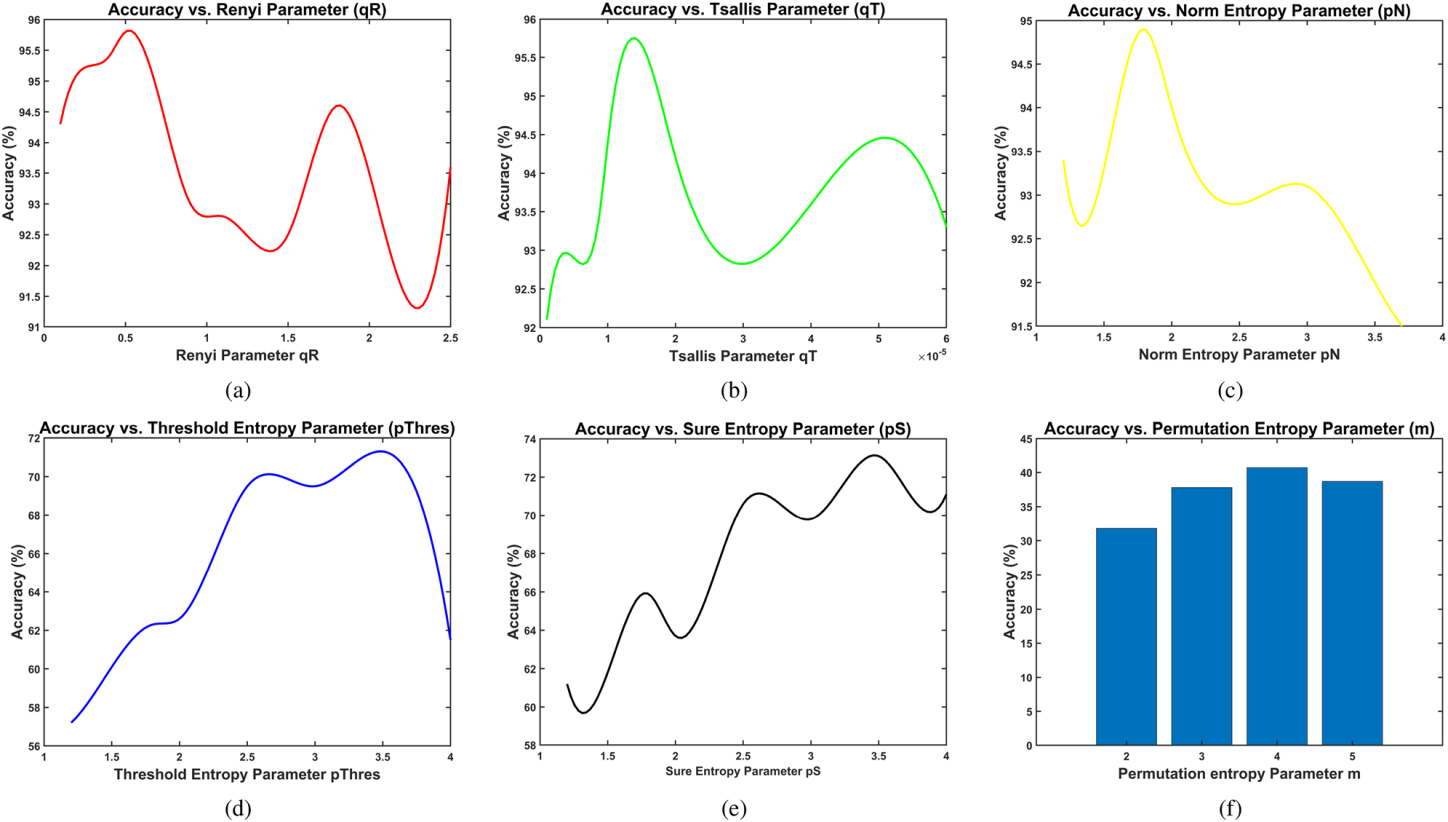
Parameters selection for the non-linear entropy feature set. The curves show the accuracy attained using HMSE for an individual feature in classifying pitting fault severity. The parameter is selected to maximize accuracy (except in the permutation entropy case). (**a**) The Rényi entropy parameter selected is $q_R=0.5$, (**b**) the Tsallis parameter is $q_T=1.5E-5$, (**c**) the norm entropy parameter is $p_N=1.8$, (**d**) the threshold entropy parameter is $p_{Thres}=3.5$, (**e**) the sure entropy parameter is $p_S=3.5$, and (**f**) the permutation entropy parameter is $m=3$.

The selection of the embedding dimension $m = 3$ was performed through an experiment comparing pitting-fault severity classification using the HMSE, considering only one feature corresponding to permutation entropy, and estimating accuracy for several values of *m*. The results are shown in Fig. 21f. Although higher accuracy is attained with $m=4$, using this value has a significant impact on computational time, so we decided to use $m=3$ as a trade-off between accuracy and efficiency. Moreover, this selection aligns with Bandt and Pompe’s original recommendation^71^. For $m = 3$, there are $m! = 6$ possible ordinal patterns, providing sufficient complexity resolution while maintaining computational efficiency and avoiding overfitting. Concerning the time lag, $\delta = 1$ was chosen because acoustic emission signals at 1 MS/s sampling rate already contain high-frequency dynamics without requiring temporal downsampling. Time lag $\delta = 1$ captures the natural temporal structure of the signal at the acquisition resolution.

#### Rényi, Tsallis, and wavelet packet entropy parameters

The order parameters $q_{R} = 0.5$ (Rényi) and $q_{T} = 1.5E-5$ (Tsallis) were selected based on an experiment where the accuracy of classification was calculated based on the HMSE for the pitting fault severity, considering each entropy feature (Rényi or Tsallis) individually. Figure 21a and b show the accuracy of each feature as a function of its corresponding parameter. The selected parameters provided the highest accuracy in the corresponding experiment. Concerning the wavelet packet entropy parameters, the norm entropy parameter $p_N=1.8$, the threshold parameter $p_{Thres}=3.5$, and the sure entropy parameter $p_S=3.5$ were estimated using, for each, an experiment similar to the one used to estimate the Rényi and Tsallis parameters. The accuracy results as a function of the parameter value are shown in Fig. 21c for the norm entropy parameter, in Fig. 21d for the threshold entropy parameter, and finally in Fig. 21e for the sure entropy parameter.

### Performance variations across fault types

Classification performance varied notably across the four fault types. Root crack severity classification achieved the highest accuracy (99.50% with CHMSE-RF), while scuffing faults showed marginally lower performance (97.33% with CHMSE-RF). These differences likely reflect the distinct physical mechanisms and acoustic emission signatures associated with each fault type.

Root cracks generate distinctive transient acoustic emissions during cyclic loading as the crack opens and closes, producing characteristic high-amplitude bursts with specific frequency content^11^. Broken teeth create even more dramatic impact events during meshing, resulting in easily distinguishable AE patterns across severity levels. In contrast, pitting and scuffing are progressive surface degradation phenomena that may produce more gradual changes in AE characteristics across severity levels.

### Machine learning model selection

Random Forests consistently outperformed support vector machines, Neural Networks, k-nearest neighbors, and decision trees across all fault types and feature extraction methods. The superior performance of RF can be attributed to several factors^110^: (1) ensemble averaging reduces overfitting and variance compared to single decision trees; (2) the bootstrap aggregating approach is particularly effective for the relatively modest dataset size (1215 samples distributed across nine classes); (3) RF naturally handles the high-dimensional feature space (16 features $\times$ multiple scales) without requiring explicit feature selection or dimensionality reduction; and (4) RF is less sensitive to hyperparameter tuning than SVM and Neural Networks.

The relatively poor performance of k-NN (87% accuracy) suggests that the feature space exhibits complex, non-spherical decision boundaries that are not well-captured by distance-based classification. The moderate performance of Neural Networks (95% accuracy) likely reflects the limited training data relative to model complexity, a known challenge for neural network approaches in industrial fault diagnosis applications where labeled data acquisition is expensive and time-consuming.

### Limitations and considerations

Several limitations of this study should be acknowledged. In particular, the experimental data were acquired from a controlled laboratory test rig under predetermined load and speed conditions^92^. Real industrial gearboxes operate under more variable and complex conditions, including fluctuating loads, temperature variations, contamination, and multiple simultaneous fault modes. The generalization of our approach to such conditions requires validation with field data.

The artificially induced faults (particularly pitting created via Electrical Discharge Machining) may not perfectly replicate naturally occurring fault progression. Natural pitting develops through repeated contact stress cycles and exhibits different morphological characteristics than EDM-induced damage. This generative process could affect the acoustic emission signatures and the classification performance^111^.

While our study examined nine severity levels per fault type, the boundaries between severity classes were predetermined based on the artificial fault creation process rather than functional performance degradation criteria. In practice, severity classification should be linked to remaining useful life predictions or functional capability assessments to support maintenance decision-making.

The study focused on single fault type classification. Real gearboxes may develop multiple concurrent fault types (e.g., pitting combined with crack initiation), and the interaction between multiple faults could produce AE signatures that differ from isolated fault conditions^112^. Extending this approach to multi-label classification scenarios represents an important direction for future research.

Finally, the computational cost of the CHMSE approach, while manageable for the selected information-entropy features, remains substantially higher than that of HMSE feature extraction. For the composite hierarchical method with three layers and four scales, the feature extraction time increases by approximately 6x compared to HMSE. This trade-off between accuracy and computational efficiency must be considered for real-time monitoring applications.

### Practical implications

The demonstrated accuracy of 99.01–99.50.01.50% for broken teeth and root crack severity classification suggests that acoustic emission monitoring with CHMSE-based feature extraction could enable reliable automated diagnostic systems for critical gearbox applications. The high true positive rates (>98% for most classes) and low false positive rates (<0.5%) indicate that this approach could substantially reduce both false alarms and missed detections compared to conventional vibration-based monitoring^18,19^.

For industrial implementation, we recommend the hierarchical multi-scale approach rather than the composite hierarchical method when computational resources are constrained or real-time processing is required. The performance difference between these methods is statistically insignificant for most fault types, while the computational cost reduction is substantial. The CHMSE approach should be reserved for applications where maximum accuracy is essential and offline processing is acceptable.

The confusion patterns observed in intermediate severity classes suggest that diagnostic systems should incorporate confidence measures and request human expert review when classification confidence falls below threshold values, particularly for classes P6-P8. Implementing a hierarchical diagnostic approach that first distinguishes healthy from faulty conditions, then identifies fault type, and finally assesses severity level may improve overall system reliability.

## Conclusions

By integrating composite hierarchical multi-scale entropy with non-linear information entropy derived from acoustic emission data, this research achieves high precision in identifying the severity of spur gearbox faults. This approach fills a significant void in current condition monitoring techniques, showcasing the robust diagnostic potential of multi-scale entropy when applied to acoustic emission signals.

The experimental investigation across four distinct fault mechanisms (pitting, broken teeth, root cracks, and scuffing) with nine severity levels each yielded several milestones. Firstly, the CHMSE approach combined with Random Forest classifiers achieved exceptional classification accuracies ranging from 97.77% for scuffing faults to 99.50% for crack faults. These results represent a substantial advancement over conventional single-scale feature extraction methods and demonstrate the viability of acoustic emission-based condition monitoring for industrial gearbox applications. Secondly, the systematic comparison of five multi-scale entropy approaches revealed that hierarchical decomposition methods (HMSE and CHMSE) consistently outperformed the CMSE approaches. The hierarchical methods preserve both low-frequency and high-frequency components of acoustic emission signals through complementary operators $Q_0$ and $Q_1$. In contrast, conventional coarse-graining effectively performs low-pass filtering that discards high-frequency fault signatures critical for early detection. Thirdly, the non-linear information entropy features demonstrated 1–4% accuracy improvements over hierarchical multi-scale statistical features across all fault types. Importantly, the selected information entropy features avoid computationally expensive vector distance calculations, enabling analysis of extended signal windows without prohibitive processing times.

In addition, the random forests consistently outperformed support vector machines, artificial neural networks, k-Nearest Neighbors, and decision trees across all experimental conditions. The superior performance of RF can be attributed to ensemble averaging that reduces overfitting, effective handling of high-dimensional feature spaces, and robustness to the relatively modest dataset size.

This research makes several principal contributions to the field of acoustic emission-based condition monitoring. The research provides the most comprehensive evaluation to date of multi-scale entropy methods applied to acoustic emission signals for fault severity classification in rotating machinery. Additionally, the integration of SHAP-based explainability analysis revealed that generalized entropy measures (Rényi entropy and Tsallis entropy) emerge as consistent primary discriminators across all multi-scale approaches, with threshold entropy and log energy entropy demonstrating substantial importance when combined with hierarchical decomposition methods (HMSE and CHMSE). These physics-informed entropy features provide transparent explanations that address a critical limitation of black-box deep learning approaches. This interpretability enables maintenance engineers to understand which signal characteristics drive diagnostic decisions, facilitating trust and adoption in safety-critical industrial applications. While previous studies have demonstrated the effectiveness of multi-scale approaches for vibration signal analysis, this work establishes that these methods are also very effective when applied to the higher-frequency, more transient nature of acoustic emission signals.

The research also demonstrates that computationally efficient information entropy features can match or exceed the performance of more complex non-linear features. The computational advantage enables practical implementation in industrial environments where processing resources may be limited and real-time decision-making is required.

While this study demonstrates promising results, several limitations must be acknowledged. The experimental data were acquired from a controlled laboratory test rig with artificially induced faults under predetermined operating conditions. Validation with naturally occurring faults in industrial environments operating under variable loads, speeds, and environmental conditions is essential before widespread deployment. Additionally, the study focused on single-fault-type classification; real gearboxes may develop multiple concurrent fault types whose interactions could produce acoustic emission signatures that differ from isolated fault conditions.

Future research should address these limitations through several specific directions, such as the investigation of optimal combinations of information entropy, statistical, and frequency-domain features to determine whether complementary information can further improve classification performance, particularly for intermediate severity classes that exhibited higher misclassification rates. It is also important to develop methods to transfer diagnostic models trained on laboratory data to field applications with different gearbox geometries, operating conditions, and sensor configurations, thereby reducing the need for extensive labeled training data in each new deployment. The development of interpretable deep learning architectures specifically designed for acoustic emission based fault severity classification. To address the trade-off between accuracy and interpretability identified in this comparison, our future research directions include the development of interpretable deep learning architectures specifically designed for acoustic emission-based fault severity classification. The aim of such research will be to explore attention-based mechanisms with entropy-informed preprocessing by combining the representational power of attention with the physics-grounded entropy features demonstrated in this study. In addition, the research should also consider explainability modules such as attention weight visualization, gradient-based saliency maps, and concept bottleneck layers that can provide physics-consistent explanations. This would allow the development of architectures that explicitly model the transition from incipient to advanced damage stages for prognostic maintenance and remaining useful life estimation.

## Data Availability

The dataset used in this research is available upon request and will be provided by the corresponding author.
